# Entry Inhibitors of SARS-CoV-2 Targeting the Transmembrane Domain of the Spike Protein

**DOI:** 10.3390/v17070989

**Published:** 2025-07-16

**Authors:** Kristin V. Lyles, Shannon Stone, Priti Singh, Lila D. Patterson, Janhavi Natekar, Heather Pathak, Rohit K. Varshnaya, Amany Elsharkawy, Dongning Liu, Shubham Bansal, Oluwafoyinsola O. Faniyi, Sijia Tang, Xiaoxiao Yang, Nagaraju Mulpuri, Donald Hamelberg, Congbao Kang, Binghe Wang, Mukesh Kumar, Ming Luo

**Affiliations:** 1 Department of Chemistry, Georgia State University, Atlanta, GA 35302, USA; kvanmouwerik1@gsu.edu (K.V.L.); psingh25@gsu.edu (P.S.); rvarshnaya@gsu.edu (R.K.V.); dliu20@gsu.edu (D.L.); sbansal4@gsu.edu (S.B.); ofaniyi1@student.gsu.edu (O.O.F.); stang13@student.gsu.edu (S.T.); xyang20@gsu.edu (X.Y.); mulpurinagaraju@gsu.edu (N.M.); dhamelberg@gsu.edu (D.H.); bwang31@gsu.edu (B.W.); 2 Department of Biology, Georgia State University, Atlanta, GA 35302, USA; sstone12@gsu.edu (S.S.); lpatterson37@gsu.edu (L.D.P.); jnatekar1@student.gsu.edu (J.N.); hpathak1@gsu.edu (H.P.); aelsharkawy2@gsu.edu (A.E.); mkumar8@gsu.edu (M.K.); 3 Center for Diagnostics and Therapeutics, Georgia State University, Atlanta, GA 35302, USA; 4 Institute for Biomedical Sciences, Georgia State University, Atlanta, GA 35302, USA; 5 Experimental Drug Development Centre (EDDC), Agency for Science, Technology, and Research (A*STAR), Singapore 138634, Singapore; kang_congbao@eddc.sg

**Keywords:** coronavirus, spike protein, transmembrane domain, antiviral, entry inhibitor, pan-coronavirus

## Abstract

Despite current vaccines and therapeutics targeting SARS-CoV-2, the causative agent of the COVID-19 pandemic, cases remain high causing a burden on health care systems. Spike-protein mediated membrane fusion of SARS-CoV-2 is a critical step in viral entry. Herein, we describe entry inhibitors identified by first screening a library of about 160 compounds and then analogue synthesis. Specifically, compound **261** was found to inhibit SARS-CoV-2 infection in a tissue model with IC_50_ of 0.3 µM. Using NMR, we found that **261** interacts with key residues in the aromatic-rich region of the spike protein directly next to the transmembrane domain. Molecular dynamic simulations of the **261** binding pocket in the spike protein was also mapped to the transmembrane domain, consistent with NMR findings. The amino acids in the binding site are conserved among different coronaviruses known to infect humans; therefore, inhibitors targeting this conserved binding site could be a useful addition to current therapeutics and may have pan-coronavirus antiviral activities.

## 1. Introduction

A critical genetic adaptation that enables zoonotic spillover and cross-species transmission of viruses is the acquisition of mutations in the viral receptor-binding protein [1]. These mutations allow the virus to recognize and bind novel receptors expressed by host cells, facilitating viral entry and infection. For severe acute respiratory syndrome coronavirus 2 (SARS-CoV-2), the causative agent of COVID-19, these adaptations were identified in the spike (S) protein, allowing recognition of the human host receptor angiotensin converting enzyme 2 (ACE2) [2]. SARS-CoV-2 is an enveloped, positive-sense, RNA virus belonging to the family *Coronaviridae* within the suborder *Coronavirineae* and order *Nidovirales*. The coronavirus virion is composed of four structural proteins: spike (S), envelope (E), membrane (M), and nucleocapsid (N), along with a number of nonstructural proteins involved in viral replication. The S-protein is a Class I viral fusion protein that protrudes from the host-derived viral envelope via its transmembrane domain (TMD), giving the virus its characteristic crown-like appearance for which it is named [3,4].

SARS-CoV-2 infection is initiated by the recognition and subsequent binding of the S-protein to the host cell surface receptor ACE2. The interaction with ACE2, together with host factors such as the host protease TMPRSS2, triggers conformational changes in the S-protein, facilitating viral entry by either direct fusion at the host membrane or receptor-mediated endocytosis. Membrane fusion is a highly regulated, multistep process that does not occur spontaneously. Several essential elements are required to initiate and complete fusion between the viral and host membranes. The S-protein is first cleaved by host proteases into two subunits: S1, which mediates receptor binding via the receptor binding domain (RBD), and S2, which contains a hydrophobic fusion peptide and facilitates membrane fusion [5]. This proteolytic cleavage can occur either during viral egress from the host cell or following endocytocis within the endosome. To initiate fusion, the fusion peptide must insert into the target membrane. However, the fusion peptide remains buried within S-protein until conformational changes are triggered within the endosomal environment. These structural rearrangements expose the fusion peptide, enabling its projection into the target membrane and disrupting the lipid order of the membrane [6]. Host membrane microdomains contain phospholipids, cholesterol, and ceramide, and are critical for membrane fusion. Cholesterol-recognition motifs within the fusion peptide are proposed to mediate interactions with specific lipid components, such as cholesterol and ceramide, which are essential for peptide-induced membrane fusion [6,7,8]. Following peptide insertion, the S2 subunit undergoes a conformational rearrangement, changing from its initial extended conformation to a more compact post-fusion conformation [9]. Interactions between the heptad repeat 1 (HR1) and heptad repeat 2 (HR2) regions stabilize the post-fusion conformation [10]. This conformational change brings the viral and host membranes into close proximity to promote fusion. Endosomal pH and ionic strength may also modulate both fusion peptide–membrane interactions and conformational changes in the S2 subunit [6,11].

The S-protein’s TMD is a critical element for viral entry. The TMD is embedded within the viral membrane and forms a trimeric helix bundle that anchors the S-protein within the lipid bilayer [12]. This domain, along with its adjacent sequences, can be divided into three functional regions: a juxtamembrane aromatic region, a central region, and the cysteine-rich region [13]. The juxtamembrane region has been shown to interact with phospholipids to facilitate membrane fusion [14]. Mutational analyses with SARS-CoV have demonstrated that substitution of key tryptophan residues in this region significantly diminishes the fusion activity [14,15]. S-protein mediated membrane fusion is a critical part of viral entry into host cells and represents a key target for antiviral intervention.

In 2003, the U.S. Food and Drug Administration approved enfuvirtide as the first therapy to inhibit the entry of human immunodeficiency virus (HIV-1) into host CD4 lymphocytes, a milestone for the development of virus entry inhibitor-based therapeutics [16]. Since then, entry inhibition by peptides or small molecule inhibitors that prevent the conformational refolding of receptor proteins has become a common approach [17]. Neutralizing antibodies that target the head of a prehairpin complex of the SARS-CoV-2 S-protein were shown to arrest the intermediate structure of the S-protein during its conformational transition, preventing fusion of the viral and target membranes [18]. The antipsychotic drug chlorpromazine (CPZ) is known to perturb membrane structure and could effectively inhibit S-protein-mediated membrane fusion by blocking insertion of the fusion peptide [6]. Another study screening a repurposed drug library found that compounds disrupting cholesterol in the plasma membrane also inhibited S-protein-mediated membrane fusion in a SARS-CoV-2 model [19]. Therefore there are several proven mechanisms for the inhibition of viral membrane fusion.

Previously we identified a class of small molecule inhibitors that bind tightly to influenza virions and prevent membrane fusion [20]. In response to the COVID-19 pandemic, we re-examined this compound library to identify inhibitors specific for SARS-CoV-2 and synthesized a series of new analogues. Here, we report that compound **261**, a thiazolidinedione, was able to inhibit SARS-CoV-2 entry. This inhibitor’s binding site was mapped using nuclear magnetic resonance (NMR) specifically at the juxtamembrane region of the TMD of the S-protein. Molecular dynamic (MD) simulation corroborated this binding site and offered insight into the possible mechanism of action. This region is highly conserved amongst coronaviruses; therefore, such inhibitors not only have the potential to contribute to pandemic preparedness, but may also exhibit broad-spectrum antiviral activity.

## 2. Materials and Methods

### 2.1. Cells and Virus Stocks

*Cercopithecus aethiops* kidney epithelial cells (VeroE6) (ATCC; CRL-1586) were cultured in Dulbecco’s Modified Eagle’s medium (DMEM) (FisherScientific, Atlanta, GA, USA; MT10027CV), supplemented with 10% heat-inactivated fetal bovine serum (FBS) and 1% penicillin–streptomycin, and incubated at 37 °C with 5% CO_2_. The ancestral Wuhan strain of SARS-CoV-2 (USA-WA1/2020) was obtained from BEI Resources (NR-52281) and propagated in VeroE6 cells. Briefly, T-25 flasks were seeded with 7 × 10^5^ VeroE6 cells in 5 mL of DMEM containing 2% FBS and 1% penicillin–streptomycin. The cells were incubated for 24 h, and then cell count was determined using a Countess 3 Automated Cell Counter (ThermoFischer, Waltham, MA, USA). At the time of infection, flasks were visually confirmed to be ~60% confluent. The media was aspirated from the flask and replaced with 5 mL of warm DMEM without additives. The initial virus stock provided by BEI was thawed at room temperature and briefly vortexed. Flasks were inoculated with 100 μL of virus and incubated for 48 h. After 48 h the supernatant was collected, aliquoted, and stored at −80 °C. To determine viral titer, 6-well tissue culture plates were seeded with 2 × 10^6^ VeroE6 cells per well in 2 mL of media. Ten-fold dilutions of the virus stock were prepared in DMEM and used to inoculate wells. Plates were then incubated for 1 h, gently swirling every 15 min. Each well was then overlayed with 2 mL of an overlay mixture composed of a 1:1 ratio of media and 2% agarose solution, and then incubated for 48 h. A secondary overlay containing 2% neutral red was applied for plaque visualization. After the final 24 h incubation, plaques were enumerated to determine viral titer. All experiments involving live SARS-CoV-2 virus were performed in a certified BSL-3 laboratory at Georgia State University.

### 2.2. In Vitro Antiviral Compound Screening

Antiviral compounds were stored at −20 °C and a concentration of 1 mM. For the assay, they were serially diluted in dimethyl sulfoxide (DMSO) to concentrations of 0.1 mM, 0.01 mM, and 0.001 mM. The in vitro screen consisted of four steps: (1) virus and select compounds co-incubated, (2) viral infection of VeroE6 cells, (3) wash to remove unbound virus, and (4) cells were incubated 24 h in fresh media with the appropriate concentration of compounds [20].

Twelve-well plates were prepared by seeding with 2 × 10^6^ VeroE6 cells per well in 1 mL of DMEM containing 2% heat-inactivated FBS and 1% penicillin–streptomycin 24 h before infection. Prior to infection, ~90% confluency was visually confirmed for each well. Previously prepared aliquots of SARS-CoV-2 were thawed at room temperature and vortexed prior to being diluted in DMEM without additives to obtain an MOI = 0.01. In a 96-well plate, 1 μL of each compound dilution was added to 99 μL of diluted virus to obtain working concentrations of 10.0 μM, 1.0 μM, 0.1 μM, and 0.01 μM compound. Experimental controls were prepared in separate wells as follows: 1 μL media (DMEM) added to 99 μL of diluted virus (Virus + Media), 1 μL DMSO added to 99 μL diluted virus (Virus + DMSO), 1 μL DMSO added to 99 μL DMEM (Media + DMSO), and 100 μL of DMEM (Media Only). The 96-well plate was then sealed and incubated at room temperature and vortexed every 15 min for 1 h.

Next, the previously prepared VeroE6 cells were infected. The media was aspirated from the cells and replaced with 150 μL of DMEM without additives. Wells were inoculated with 100 μL of either virus + compound dilutions or control mixtures. Plates were gently rocked every 15 min for 1 h to ensure the distribution of the diluted compounds or control mixtures over the cells and incubated at 37 °C with 5% CO_2_. Compound dilutions or control mixtures were then aspirated from wells. Wells were washed by gently rocking the plates with 200 μL of warm 1X PBS added to each well before being carefully aspirated from each well. Next, 990 μL of DMEM containing 2% FBS and 1% penicillin–streptomycin was subsequently added to each well along with 10 μL of the corresponding compound dilution or control solution. Plates were incubated for 24 h.

Supernatant was then collected and stored at −80 °C for later analysis. The cells were washed with PBS as described previously. Then, 360 μL of RLT-βME lysis buffer was added, swirling to ensure full coverage of the well before allowing the plate to sit for 2 min at room temperature. Plates were tilted forward to allow pooling of lysis buffer, which was then vigorously pipetted over the well. Cell lysate was then collected and stored at −80 °C for later analysis. Repeats were run for compounds that displayed dose-dependent inhibition (i.e., Class I or Class II compounds) to confirm observed inhibition, while compounds that failed to show inhibition (i.e., Class III) or were observed to be cytopathic were excluded from further studies.

### 2.3. RNA Extraction and Quantitative rtPCR

Total RNA was extracted from cell lysate samples using the Qiagen RNeasy Mini Kit (Qiagen, Hilden, Germany, cat# 74104) and quantified using a NanoDrop microvolume spectrophotometer (ThermoScientific). Viral RNA levels were quantified via RT-qPCR using the TaqPath 1-Step RT-qPCR kit (Applied Biosystems, Waltham, MA, USA, A15300) with primers and probes specific for the SARS-CoV-2 nucleocapsid (N) gene (Qiagen. 222015, 2019-N1-Forward Sequence GACCCCAAAATCAGCGAAAT, 2019-N1-Reverse Sequence TCTGGTTACTGCCAGTTGAATCTG) [21]. Viral genome copies were then quantified through comparison to a standard curve generated using a known amount of RNA extracted from SARS-CoV-2 samples previously titrated, as described previously [22,23]. The inhibitor quantification was then calculated as a percent of the Virus + DMSO quantity and reported as relative infectivity (% virus + DMSO). Graphs displaying results were prepared using GraphPad Prism 10.

### 2.4. Characterization of Thiol Reactivity via Singlet Oxygen Detection Assay

The photosensitization activity of compounds 119, 192, and 261 was evaluated using the Singlet Oxygen Sensor Green Kit (ThermoFisher) Briefly, each compound was incubated with Singlet Oxygen Sensor Green in a solvent mixture of acetonitrile (ACN) and Tris buffer (pH 7.4) at a 9:1 ratio. Fluorescence was measured at 525 nm (excitation = 488 nm) for 20 s using a Shimadzu RF-5301pc fluorescent spectrometer. Samples were exposed to visible light from a fixed-intensity cold LED light source for 30 s, followed by a second fluorescence signal measurement for 20 s. This light exposure and measurement cycle was repeated seven times. Fluorescence intensity was plotted against time to assess singlet oxygen production using GraphPad Prism 10.

### 2.5. Purification of S-TMD

The cDNA encoding the transmembrane domain of the SARS-CoV-2 spike (S) protein (amino acids 1201–1239; referred to as S-TMD) was cloned into the *NdeI* and *XhoI* restriction sites of the pET15b plasmid [12]. Briefly, the resulting plasmid construct encoded a recombinant protein consisting of the S-TMD region fused to an N-terminal tag: MGSSHHHHHHSSGLVPRGS. The plasmid was transformed into *Escherichia coli* (*E. coli*) BL21 (DE3) competent cells for recombinant protein expression. Transformed cells were cultured in 1 L of M9 medium supplemented with 100 µg/mL ampicillin. When the culture reached an optical density at 600 nm (OD_600_) of 0.6–0.8, protein expression was induced with 1 mM isopropyl β-D-1-thiogalactopyranoside (IPTG), followed by incubation at 37 °C and 200 rpm overnight. S-TMD was purified and resuspended in a buffer containing 20 mM sodium phosphate (pH 7.2), 1.56% dihexanoylphosphatidylcholine (DHPC), and 0.44% 1,2-dimyristoyl-sn-glycero-3-phosphocholine (DMPC). The final protein sample was concentrated to 0.1–0.5 mM and stored at −80 °C for downstream applications.

### 2.6. NMR Experiments

To assign the ^1^H-^15^N-HSQC spectrum, a uniformly ^13^C/^15^N-labeled S-TMD sample was prepared for data collection. The following experiments including 2D ^1^H-^15^N-HSQC, 3D-HNCACB, HNCA, HNCO, and HNCOCA were collected on a Bruker 600 MHz magnet at 40 °C. Spectral assignment was performed based on the previously published assignments in micelles and the collected data sets [12]. For this study S-TMD was incorporated into bicelles at a concentration of 0.4 mM. The test compound was dissolved in DMSO to obtain a 40 mM stock solution. The ^1^H-^15^N-HSQC spectra of S-TMD in the presence of 4% DMSO alone and 1.6 mM compound **261** in 4% DMSO were collected and the ^1^H-^15^N-HSQC spectra with and without compound **261** where processed, visualized, and then compared to assess ligand binding.

### 2.7. Computation Methods for Inhibitor–Protein Complexes

#### 2.7.1. System Preparation

Two trimeric models of SARS-CoV-2 spike transmembrane domain (TMDTri) were constructed, the first model using the AlphaFold 3.0 server [24] and the second using the SWISS-MODEL server [25] in conjunction with NMR data. A sequence of 40 residues (sequence: MQELGKYEQYIKWPWYIWLGFIAGLIAIVMVTIMLSSMTS) was used for each monomer. A total of eleven membrane-bound systems of TMDTri were prepared using the Membrane Builder Module of CHARMM-GUI [26,27]: one without ligands, and ten with ligands in different orientations and at different positions of TMDtri models. Dimyristoylphosphocholine (DMPC) lipids were used to construct the lipid-bilayer. Each ligand-bound system contains three ligands in the same orientation. In conf-1, the furan’s oxygen atom is oriented toward the protein, while in conf-2, it is directed toward the lipid bilayer. During the construction of the simulation box at pH 7.0, water molecules were added with a thickness of 40 Å from protein/bilayer along the Z-axis. The final simulation box dimensions were 86 Å × 86 Å × 131 Å. The ligand-free system contains 204 DMPC lipid molecules and 20,561 water molecules, while the ligand-bound system has 203 DMPC lipid molecules and 20,474 water molecules. These systems were neutralized with sodium and chloride ions and used for molecular dynamics (MD) simulations [28].

#### 2.7.2. Molecular Dynamics Simulations

MD simulations were conducted using the AMBER 22 software suite [29], incorporating the AMBER ff19SB force field for protein [30]. The DMPC lipid molecules were parameterized using the Lipid21 force field [31], while the water molecules were modeled with the TIP3P water model [32]. The ligand molecules were assigned GAFF2 parameters [33] with AM1-derived charges [34].

The energy minimization process involved 5000 steps: 3000 steps using the steepest descent algorithm followed by 2000 steps employing the conjugate gradient method. During minimization, harmonic positional restraints were applied to the solute atoms, gradually reducing the force constant from 500 to 0 kcal·mol^−1^·Å^−2^ over five stages. The systems were then heated from 100 K to 300 K under NVT conditions using a Langevin thermostat with a collision frequency (γ) of 1.0 ps^−1^ and a 500 ps timescale. A time step of 1 fs was used, and positional restraints on the solute were progressively reduced over five steps (500, 300, 100, 50, and 5 kcal·mol^−1^·Å^−2^). The system was heated for 1 ns under NPT conditions (300 K, 1 bar) using a 1 fs time step. Positional restraints on the solute were gradually reduced over eight steps (500, 300, 100, 50, 10, 2, 1.0, and 0.5 kcal·mol^−1^·Å^−2^). A Monte Carlo barostat with a coupling constant (τp) of 1.0 ps was applied, with pressure controlled along the z-axis. The system was equilibrated for 20 ns under NPT conditions (300 K, 1 bar) using a 2 fs time step. Electrostatic interactions were managed using the particle-mesh Ewald (PME) method [35], while a 9.0 Å cutoff was set for short-range nonbonded interactions. Hydrogen-involving bonds were constrained using the SHAKE algorithm [36], and simulation snapshots were recorded every 10 ps.

The production phase of each simulation extended to 1.0 μs, with and without ligands, resulting in a cumulative simulation time of 11.0 μs. During a 1.0 μs simulation, the ligand remained bound to TMDtri in only two out of ten ligand-bound systems. For analysis, 1.0 μs of each trajectory was considered to evaluate the conformational characteristics of TMDtri. Structural stability and flexibility were assessed using root-mean-square deviations (RMSD) and radius of gyration (Rg) calculations. The RMSD was computed for backbone atoms (N, Cα, C, and O), while the Rg was calculated using all heavy atoms. All analyses were performed using CPPTRAJ from AmberTools [37]. Prior to analysis, all frames were aligned to the first frame of the trajectory based on backbone atoms.

#### 2.7.3. Binding Free Energy Calculations

Binding free energies were computed using the MMPBSA module in Amber 22 [38,39]. The production run trajectory was post-processed for analysis with CPPTRAJ to remove solvent, membrane, and counterions from the receptor–ligand complex. A total of 100,000 frames, extracted from a 1 µs simulation of each trajectory, were analyzed to calculate molecular mechanics potential energies and solvation free energies. The binding free energy of the protein–ligand complex was determined by subtracting the combined free energies of the isolated receptor and ligand from that of the complex in the membrane protein–ligand system [38].

(1) $${∆G}_{bind,solv}={∆G}_{bind,vac}+{∆G}_{complex,solv}-\;{∆G}_{receptor,solv}-\;{∆G}_{ligand,solv}$$

where ${∆G}_{complex,solv},{∆G}_{receptor,solv},{and∆G}_{ligand,solv}$ are solvation free energies of complex, receptor, and ligand, respectively.

Solvation free energies were calculated using the following formula:

(2) $${∆G}_{sol}={∆G}_{solv,\;polar}+{∆G}_{solv,\;nonpolar}$$

The polar component (${∆G}_{solv,\;polar})$ was determined by solving the Poisson–Boltzmann equation, while the nonpolar term $({∆G}_{solv,\;nonpolar})\;$was calculated using the classical model. The computed binding free energies were subsequently compared with experimental data for validation.

## 3. Results

### 3.1. SARS-CoV-2 Entry Inhibitor Screening

Work by Rowse et al. identified viral entry inhibitors that bind the influenza virion and prevent lipid mixing [20,40]. We identified compounds from this library that had a modest inhibition against Influenza and screened for inhibition of SARS-CoV-2 infections. In total, we screened 168 compounds from the influenza library at four different concentrations (10.00, 1.00, 0.10, and 0.01 µM). Compounds were incubated with virus for an hour before infection, and then an hour after infection the cells were washed and provided fresh media with the appropriate compound. Compounds that showed a 20% reduction in virus yield in comparison with Virus + DMSO were repeated to confirm inhibition. Based on these results the compounds were grouped in three classes: Class I, 50% inhibition at a compound concentration of <0.35 µM; Class II, 50% inhibition at a compound concentration of 0.35 < 5.00 µM; and Class III, no inhibition or lower potency (92 compounds).

The Class I and Class II inhibitors provided a glimpse into the structure–activity relationship (SAR) of these compounds (Table 1, Supplementary Materials). The compounds have a central scaffold, with modifications of the R_1_ and R_5_ groups. For Class I compounds, R_1_ needs to have a hydrophobic group like bicycloheptane. This type of R_1_ group is likely to render the compound strong hydrophobic interactions with the target. The R_5_ group is a benzyl ring with various modifications of the aromatic ring with small substituents. This type of R_5_ group can also have hydrophobic interactions with the target, while having potential polar interactions by the small substituents. Modifications of the R_4_ moiety were also found, which may offer polar interactions by hydroxyl or amino ends. In general, only selected combinations of specific modifications are required for potency.

One of the major differences for Class II compounds in the R_1_ group is adamantane in place of bicycloheptane. Adamantane is bulkier than bicycloheptane and may therefore not fit well in the interaction site with the target. The introduction of a carboxyl group to modify the R_4_ or R_5_ group also reduced the potency of the compound.

Modifications in Class III compounds are more diverse, bulkier, and include groups with large sizes or charges. No clear trend can be identified.

### 3.2. Analogue Synthesis

**Scheme 1:**
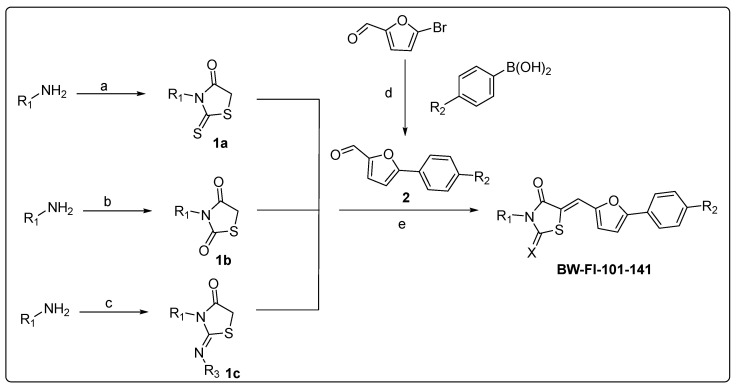
Synthesis of new analogs. Reagents and conditions: (a) sodium chloroacetate, CS_2_, Et_3_N, EtOH; (b) thioglycolic acid, CDI, DIPEA, DCM, 12 h, rt, 60–88%; (c) (i) chloroacetyl chloride, Et_3_N, dry DCM, 30–81%, (ii) KSCN, dry acetone, 65 °C, 3 h, 30–81%, (iii) CsCO_3_, rt, 1h; (d) Pd(Ph_3_)_4_, K_2_CO_3_, dioxane/water, reflux, 6h, 55%; (e) piperidine, EtOH, 54–94%.

**Scheme 2:**
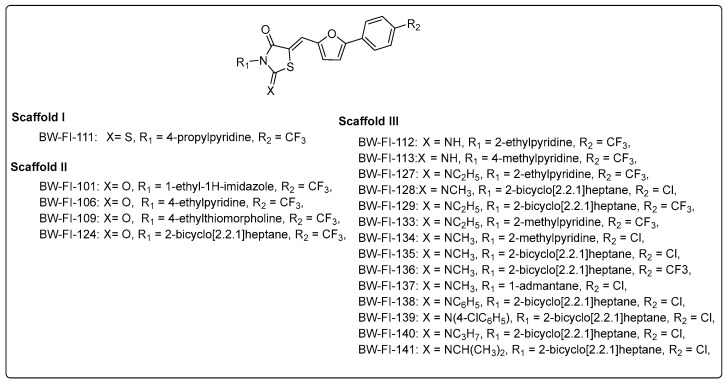
Analog structures in scaffolds I, II, and III.

To explore possible improvements to inhibition, a number of analogs were synthesized. The new compounds (**BW-FI-101-141**) were synthesized, as shown in Scheme 1 and Scheme 2. The first step involved the five-membered ring (**1a**, **1b,** and **1c)** formation using different procedures (see Supporting Information). Substituted furan aldehyde (**2**) was synthesized by the Suzuki coupling reaction and reacted with compounds **1a**, **1b,** and **1c** to obtain final compounds (**BW-FI-101-141**), respectively. The yield of the final compounds was between 52 and 93%.

### 3.3. Chemical Characterization of Compound 261

**Figure 1:**
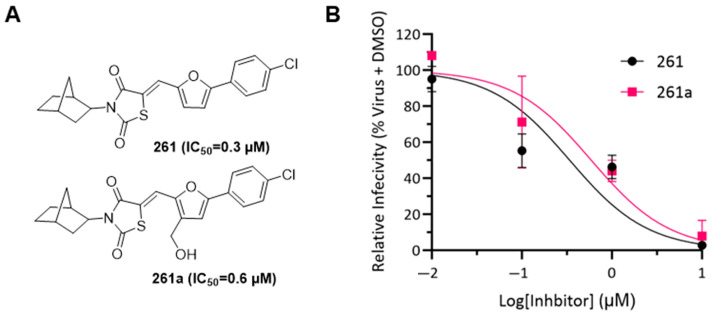
Determining IC_50_ for compounds **261** and **261a**. (**A**) Structure of **261** and **261a** with corresponding IC_50_ data; note the difference is the incorporation of a hydroxyl substitution on the furan ring. (**B**) SARS-CoV-2 entry inhibition assay was performed using a monolayer of VeroE6 cells. For compound **261** (black circles), the IC_50_ value was found to be 0.3 µM and the IC_50_ value for **261a** (pink squares) was 0.6 µM. The measurements were carried out in triplets and error bars are included in the graph.

Compound **261** (Figure 1A) was identified by our initial screen of existing compounds as a Class I inhibitor with an IC_50_ of 0.3 μM (Figure 1B). It was selected for further studies because it belongs to a family of drug compounds known as thiazolidinediones (glitazones). Thiazolidinediones are insulin sensitizers and have been used as medication for type 2 diabetes [41]. Belonging to the thiazolidinedione group, **261** is likely to have acceptable drug-like pharmacological properties. To explore further the binding pocket, compound **261a** (Figure 1A) was synthesized to incorporate a hydroxyl substitution in the furan ring and had an inhibitory effect with an IC_50_ of 0.6 μM (Figure 1B).

**Figure 2:**
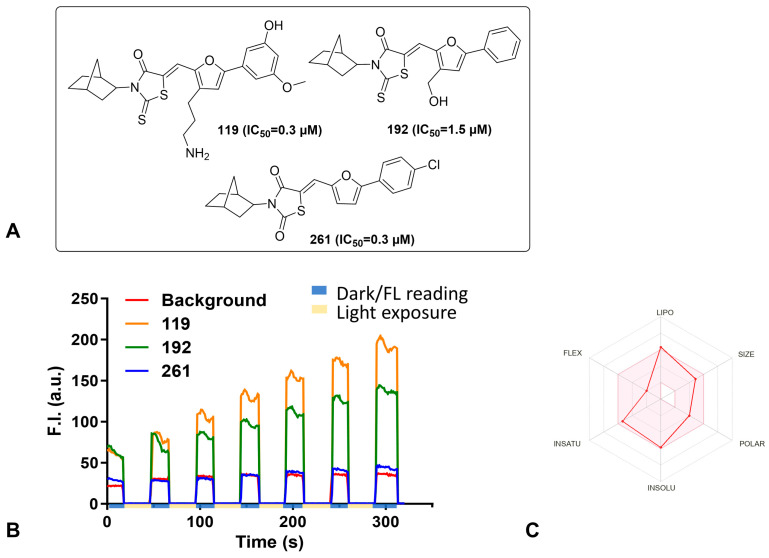
Chemical characterization of selected inhibitors. (**A**) Chemical structure of the inhibitors tested in the thiol reactivity and photosensitivity assays along with their IC_50_ concentration. (**B**) Singlet Oxygen Sensor kit. Selected compounds were exposed to light for 20 seconds (beige blocks on time axis), and then the fluorescence was measured for 30 seconds in the dark (blue blocks on time axis) for a total of 6 rounds. The fluorescent intensities were plotted as the area under the curve for each inhibitor; **119** (orange) and **192** (green) show increasing fluorescence intensity as time and light exposure progressed. In comparison, **261** (blue) did not exhibit increased fluorescence over time and instead fluorescence intensity remained similar to background readings (red) for the duration of the experiment. (**C**) The bioavailability radar plot for compound **261** (red line) as calculated by SwissADME. The area in pink is considered to have properties that are of a drug-like nature.

Michael acceptors are a concern in drug design since these compounds may form nonspecific covalent conjugations with other molecules by Michael addition. Compound **261** contains an α, β unsaturated ketone motif that could potentially be a Michael acceptor. We examined the thiol reactivity of **261** along with compound **119,** which similarly contains a thiol group but contains an amino substitution in the furan ring and different substitutions on its benzene ring (Figure 2A). A 50 µM concentration of either **261** or **119** was incubated with 1.0 mM cysteine or 1.0 mM glutathione in PBS:DMF 1:1 solution at 37 °C for 18 h. At the end of incubation, the samples were analyzed by Agilent 110 HPLC. No notable reaction or adduct formation was observed. This analysis confirmed that these compounds do not act as a reactive Michael acceptor that forms conjugates.

Since most of the compound handling occurred in the presence of light, the photosensitizer properties **261** were tested using the Singlet Oxygen Sensor kit from Thermo-Fisher (Figure 2B). Compounds **119** and **192** were selected as controls to confirm that the inhibitor effects of **261** were intrinsic to the compound and not derived from photosensitivity of the compound. The compounds were exposed to cold light at a wavelength of 488 nm for 20 seconds, and then the fluorescence of the samples was measured at 525 nm for an interval of 30 seconds. The light exposure and fluorescence measurement were repeated for six more rounds. The total fluorescence intensity after each exposure for **119** and **192** increased, indicating that they are photosensitive. Whereas, **261** has little to no measurable photosensitivity as its fluorescence intensity did not increase with increased light exposure. These results indicate that the antiviral activity of **261** is not derived from possible photosensitivity.

Other chemical properties, including lipophilicity, polarity, and solubility, are related to the pharmacological behavior of a compound. We therefore examined **261** using SwissADME, a website that computes physicochemical properties and provides a bioavailability radar plot with key parameters related to bioavailability of a compound (Figure 2C) [42]. The pink area in the radar plot represents the optimal range for each parameter. Based on this calculation, the parameters related to bioavailability of **261** are within the acceptable range, suggesting this compound has drug-like potential.

### 3.4. NMR Structural Characterization of Inhibitor–Transmembrane Domain Interactions

**Figure 3:**
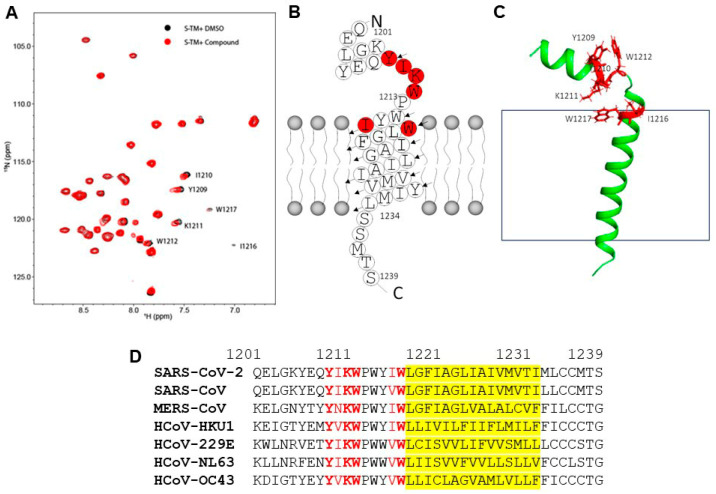
Molecular interactions between compound 261 and S-TMD by NMR spectroscopy. (**A**) The ^1^H-^15^N-HSQC spectra of S-TMD in the absence (black) and presence (red) of compound **261**. The spectra of 0.4 mM S-TMD in the presence of DMSO and 1.6 mM compound **261** were collected, processed, and superimposed. Residues exhibited chemical shift perturbations are labeled with residue name and sequence number. (**B**) Amino acid sequence and its secondary structure in membrane. Residues affected by compound binding are highlighted in red. (**C**) The affected residues of S-TMD upon compound binding. One structural model of S-TMD was plotted and the affected residues upon compound binding are shown in sticks and highlighted in red. The gray box represents the lipid membrane. (**D**) Sequence alignment of coronavirus TMD. The TMD amino acid sequences from each coronavirus that infects humans were aligned. The virus name is labeled on the left. The residue numbers correspond to that of SARS-CoV-2 spike protein. The red letters refer to amino acids with perturbation of NMR chemical shifts upon ligand binding, with strictly conserved amino acids bolded. The region covered by the fatty acid tails of the lipid bilayer are in yellow.

Using NMR, the structure of the TMD (amino acids 1209–1236) of the S-protein was determined to form trimers [43]. Our construct encompassed the TMD and the upstream juxtamembrane aromatic region (S-TMD, amino acids 1201–1239). Structural studies using this constructed in detergent micelles revealed that S-TMD peptide consists of two helices: one helix extending into the virion’s membrane and the other extended into the external space, separated by a proline break [12]. A structural model of **261** bound to S-TMD was generated based on dihedral angle restraints determined in micelles. However, the relative orientation of the helices could not be resolved due to the absence of long-range restraints. To investigate the interaction of **261**, the S-TMD was reconstituted in bicelles composed of dihexanoylphosphatidylcholine (DHPC) and 1,2-dimyristoyl-sn-glycero-3-phosphocholine (DMPC), which better mimic the membrane environment. The spectra of S-TMD in bicelles were similar to those in DPC micelles. To probe molecular interactions, ^1^H-^15^N-HSQC spectra of S-TMD were collected in the presence of DMSO and compound **261** (Figure 3A). Upon addition of compound **261**, residues Y1209, I1210, K1211, and W1212 showed chemical shift perturbations, while I1216 and W1217 exhibited line broadening. These results suggest direct molecular interactions between S-TMD and **261**. The location of these residues in relation to the S-TMD is highlighted in a red cartoon representation (Figure 3B) and ribbon model (Figure 3C). I1216 and W1217 are located just in the lipid membrane, while residues Y1209, I1210, K1211, and W1212 are located in a flexible region in between two α-helices. This region is highly conserved across human coronaviruses (Figure 3D).

### 3.5. Compound **261** Binding Site Mapped by Molecular Dynamics Simulation

**Figure 4:**
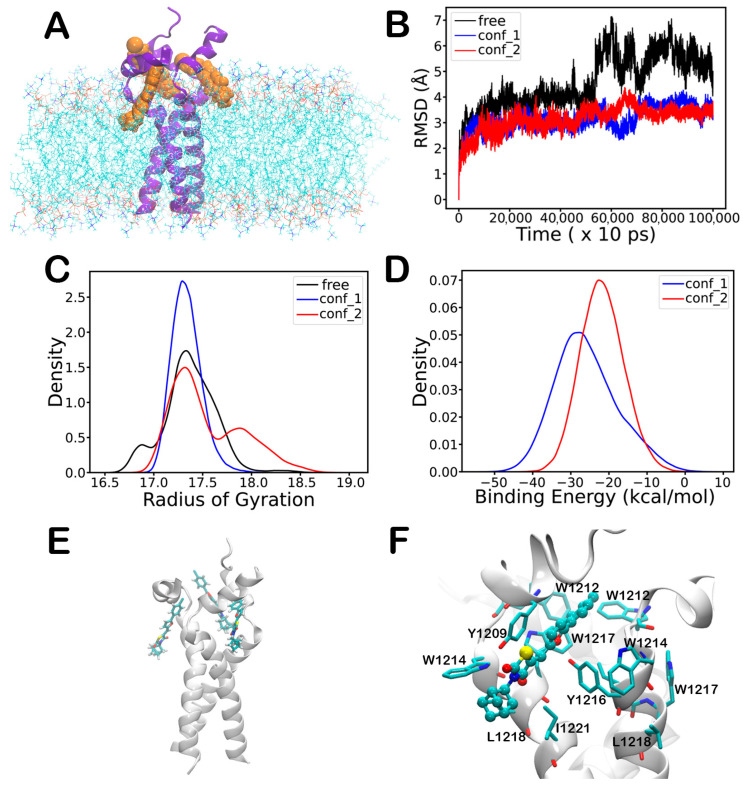
Complex formation of TMDtri with the ligand molecules obtained from 1 µs MD simulations. (**A**) TMDtri binding to three ligand molecules in DMPC lipid bilayer (water molecules removed for clarity); tri-cyclic ring of the ligand is embedded in the lipid bilayer and chloride atom exposed to solvent molecules. (**B**) RMSD of TMDtri backbone atoms in absence (black) and presence of ligands in conf_1 (blue) and conf_2 (red) orientations. (**C**) Radius of gyration of all heavy atoms with and without ligands (free—black, conf_1—blue, conf_2—red). (**D**) Binding energy of three ligands with TMDtri in conf_1 (blue) and conf_2 (red) orientations. (**E**) Interaction of three ligands with TMDtri, where all ligands are oriented with the furan oxygen atom facing the protein (conf_1). (**F**) Close-up view of a single ligand (ball and stick model) bound to TMDtri (residues in stick model with one letter code).

Two trimeric models of SARS-CoV-2 TMDTri were constructed. The stability of TMDtri was evaluated in the presence and absence of ligands by analyzing RMSD and Rg values over a 1µs MD simulation. The ligand-bound system contains 203 DMPC lipid molecules and 20,474 water molecules (Figure 4A). TMDtri exhibits greater flexibility than its ligand-bound complexes (Figure 4B). This increased flexibility may serve as a driving force for TMDtri’s activity to facilitate membrane fusion compared to the more rigid TMDtri–ligand complex. Further, the stability of the protein in different states was assessed by analyzing the Rg distributions (Figure 4C). The kernel density estimation (KDE) plots illustrate the compactness and flexibility of the protein in its free and ligand-bound conformations. Ligand binding between two neighboring helixes of TMDtri in conf_1 state exhibits the lowest Rg values with a sharp and narrow distribution, suggesting a compact and stable conformation. In the absence of ligands, TMDtri shows a broader distribution with slightly higher Rg, indicating an increased flexibility. Similarly, ligand binding of TMDtri in the conf_2 state has the highest Rg values and a more extended distribution, reflecting a more flexible conformation. The ligand binding induces compaction of the protein, stabilizing its conformation, whereas the unbound and highly flexible states may be crucial for its activity.

The binding energy distributions of ligands interacting with TMDtri in conf_1 and conf_2 orientations are presented in Figure 4D. The analysis reveals that conf_1 exhibits a stronger binding affinity, as indicated by its lower binding energy values, suggesting more stable ligand–protein interaction. In contrast, conf_2 shows higher binding energy values, reflecting a weaker binding affinity and an increased flexibility in the ligand binding interface. These results indicate that the ligand preferentially binds to TMDtri in the conf_1 orientation. The phenyl ring has π-interactions with the sidechain of Y1209 from the neighboring helix. The furan ring and the thiazolidine-2,4-dione moiety stack with the sidechain of W1217, while the thiazolidine-2,4-dione moiety also interacts with the sidechain of Y1209. These interactions are consistent with the perturbation of NMR chemical shifts upon ligand binding. The tri-cyclic ring of the ligand is embedded partially in a hydrophobic indentation lined by sidechains of W1215, L1218, and I1221 and partially in the lipid bilayer, whereas the chloride atom is exposed to the solvent molecules. Ligand perturbation was observed for NMR chemical shifts of W1215, but not of L1218 and I1221. The binding mode of the three ligands with TMDtri is presented in Figure 4E, with a close-up view shown in Figure 4F.

## 4. Discussion

Membrane fusion is energy favorable but has high kinetic barriers due in large part to repulsive hydration forces. Upon SARS-CoV-2 fusion peptide insertion into the target membrane, the H2 domain folds back, bringing the fusion peptide and the TMD regions into close proximity [11]. This brings the target and viral membranes together, allowing lipid mixing. The S-TMD is required for viral and target membrane fusion, with key amino acids located in the aromatic-rich region of the juxamembrane [13]. Cryo-electron microscopy of the post-fusion S-protein suggests that the fusion peptide serves as a docking site for the TMD in the final stages of the membrane merger, with key interactions at W1212, W1217, and Phe1220 [11]. Mutations of aromatic residues (tyrosines and tryptophans) in the S-TMD of SARS-CoV severely reduce S-mediated membrane fusion [14,15]. S-protein mediated membrane fusion is a critical part of viral entry into host cells and represents a key target for antiviral intervention.

Compound **261,** a thiazolidinedione, inhibited SARS-CoV-2 in an antiviral assay with an IC_50_ of 0.3 µM. Currently, two thiazolidinediones (rosiglitazone and pioglitazone) are approved for used by the US Food and Drug Administration for the treatment of type 2 diabetes [41]. SwissADME confirmed that the pharmacokinetic properties of **261** are within the acceptable range and suggest that it has drug-like potential. A thiol reactivity assay showed the α, β unsaturated ketone motif is not a reactive Michael acceptor, as it did not form conjugates nor was the compound photosensitive. Both are important when designing a novel drug since chemical changes can potential cause adverse effects in patients. While **261** did not exhibit cytopathic effects after incubation with VeroE6 for 24 h, more work needs to be performed on the pharmacokinetics to define toxicity and to explore metabolics and tissue distribution. One of the problems in drug design is ensuring a concentrated dose reaches the target tissue, and this is especially difficult in respiratory infections. We synthesized **261a** with a hydroxyl substitution in the furan ring, providing a handle for the preparation of a prodrug version that may enhance distribution to the lung tissue, though this did slightly reduce the potency of the compound. Future work focused on pharmacokinetics, metabolism, and tissue distribution will help us better understand how to optimize dosage and delivery.

Compound **261** is analogous with structures shown to inhibit membrane fusion of influenza virus by intercalating the viral envelope [20,40]. We hypothesized that **261** adopts the same mechanism to inhibit SARS-CoV-2 viral entry. To decipher how **261** interacts with the viral envelope, the NMR spectra were evaluated using S-TMD embedded in bicelles. The NMR data for **261** mapped the binding site to in between two neighboring helixes in S-TMD and was corroborated with an MD simulation. Compound **261** interacts with the aromatic sidechains in S-TMD and has a hydrophobic moiety immersed in the fatty acid tails of the lipid bilayer. This region was shown to be required for S-mediated membrane fusion [14,15,19]. The binding site for **261** is lined with the hydrophobic residues that are highly conserved across human coronaviruses, offering the possibility for using S-TMD as a target for developing pan-coronavirus antivirals. Our MD simulation shows that binding of compound **261** to S-TMD decreased its flexibility in the conf_1 state, suggesting this as the mechanism for **261** inhibition of membrane fusion. Yet this region in S-TMD was shown to potentially engage cholesterol in the target membrane to facilitate membrane fusion [19]. Occupation of this site by **261** would prevent its engagement with cholesterol to inhibit membrane fusion, presenting an alternative mechanism of action that should be explored in future work.

## 5. Conclusions

By screening a library of small molecules and through the synthesis of novel compounds, we identify compound **261** as an entry inhibitor for SARS-CoV-2. Through mechanistic studies, we defined the binding site of **261,** finding that it represents a unique series of SARS-CoV-2 entry inhibitors. Compound **261** binds the spike protein at the TMD and prevents viral entry of VeroE6 cells. The amino acids identified as having direct interaction with **261** through NMR and MD are highly conserved among coronaviruses that are known to infect humans and have been shown to be critical for membrane fusion in SARS-CoV. This antiviral mechanism of binding the S-TMD has the potential to treat COVID-19 and for the development of pan-coronavirus antivirals.

## Figures and Tables

**Table 1:** Common structures of active inhibitors (Class I and II) from screening.

Structure	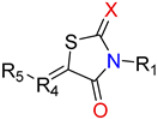			
	R_1_	X	R_4_	R_5_
Class I (23)	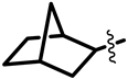	S	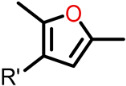 R’ = H or	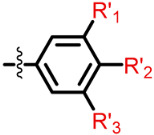 R’_1_, R’_2_, R’_3_ = H, F, Cl, OH, OCH_3_
	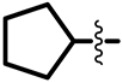	O	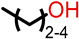 or 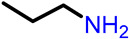	
Class II (53)	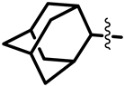		R’= 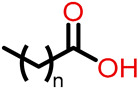	R’ = 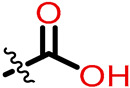

## Data Availability

The data that supports the findings of this study are available from the corresponding Author, M.L., upon reasonable request.

## Supplementary Materials

The following supporting information can be downloaded at: https://www.mdpi.com/article/10.3390/v17070989/s1, Chemical Synthesis.

## Abbreviations

The following abbreviations are used in this manuscript:

ACE2	Angiotensin converting enzyme 2
ACN	Acetonitrile
CO_2_	Carbon dioxide
Conf-1	MD simulation with **261** furan’s oxygen is orientated toward the protein
Conf-2	MD simulation with **261** furan’s oxygen is directed toward the lipid bilayer
DHPC	Dihexanoylphosphatidylcholine
DMEM	Dulbecco’s modified eagle medium
DMPC	1,2-dimyristoyl-sn-glycero-3-phosphocholine
DMSO	Dimethyl sulfoxide
E	Envelope
*E. coli*	*Escherichia coli*
FBS	Fetal bovine serum
HP1	Heptad repeat 1
HP2	Heptad repeat 2
HPLC	High-performance liquid chromatography
ITPG	Isopropyl β-D-1-thiogalactopyranoside
KDE	Kernel density estimation
LED	Light-emitting diode
M	Membrane
MD	Molecular dynamics
MOI	Multiplicity of infection
N	Nucleocapsid
NMR	Nuclear magnetic resonance
OD_600_	Optical density at 600 nm
PME	Particle-mesh Ewald method
RBD	Receptor binding domain
Rg	Radius of gyration
RMSD	Root-mean-square deviations
rtPCR	Reverse transcription polymerase chain reaction
S	Spike
S1	Region of spike protein that mediates receptor binding
S2	Region of spike protein that facilitates membrane fusion
SAR	Structure–activity relationship
SARS-CoV-2	Severe acute respiratory syndrome coronavirus 2
S-TMD	Elongated transmembrane domain construct used in our study
TMD	Transmembrane domain
TMDtri	Trimer of transmembrane domain
VeroE6	*Cercopithecus aethiops* kidney epithelial cells
Τp	Coupling constant
