# Supplementary material for: Entry Inhibitors of SARS-CoV-2 Targeting the Transmembrane Domain of the Spike Protein
Source: Viruses. 2025 Jul 16;17(7):989. doi: 10.3390/v17070989 (PMC12300635; doi:10.3390/v17070989)
Supplement: Supplementary file 1 [file viruses-17-00989-s001.zip › viruses-3703745-supplementary.pdf]

Supporting Information for

Entry inhibitors of SARS-CoV-2 Targeting the Transmembrane Domain of the Spike Protein

Kristin V. Lyles[1], Shannon Stone[2], Priti Singh[1,3], Lila D. Patterson[2], Janhavi Natekar[2], Heather Pathak[2], Rohit K. Varshnaya[1], Amany Elsharkaway[2], Dongning Liu[1], Shubham Bansal[1,3], Oluwafoyinsola O. Faniyi[1,3], Sijia Tang[1,4], Xiaoxiao Yang[1,3], Nagaraju Mulpuri[1], Donald Hamelberg[1,3], CongBao Kang[5], Binghe Wang[1,3], Mukesh Kumar[2,3], and Ming Luo[1,3,]*

[1]Department of Chemistry, Georgia State University, Atlanta, GA 35302, USA

[2]Department of Biology, Georgia State University, Atlanta, GA 35302, USA

[3]Center for Diagnostics and Therapeutics, Georgia State University, Atlanta, GA 35302, USA

[4]Institue for Biomedical Sciences, Georgia State University, Atlanta, GA 35302, USA

[5]Experimental Drug Development Centre (EDDC), Agency for Science, Technology, and Research (A*STAR), Singapore 138634

*to whom correspondence and material requests should be addressed. Email: mluo@gsu.edu

1. Chemical synthesis

General Information-:

All solvents were of reagent grade and purchased from Fisher Scientific. All chemicals were of reagent grade and purchased from Sigma-Aldrich (Massachusetts, USA), Ambeed, Inc.

(Illinois, USA), or Oakwood Products, Inc. (South Carolina, USA). Column chromatography was carried out using silica gel (230 – 400 mesh) (Sorbent Technologies, Georgia, USA). TLC analyses were conducted on Silica XHL TLC plates w/UV254 (Sorbent Technologies, Georgia, USA). ^{1}H NMR (400 MHz) and ^{13}C NMR (100 MHz) spectra were recorded on a Bruker Avance 400 MHz NMR spectrometer in deuterated solvent from Oakwood Products, Inc. Chemical shifts were reported as δ values (ppm). TMS (δ = 0.00 ppm) or residual peaks of the deuterated solvent were used as the internal reference. Mass spectrometric analyses were conducted at the Georgia State University Mass Spectrometry Facilities. Samples of milligram quantities were weighed on a C-33 microbalance for bioassays (CAHN Instruments Inc., California, USA). A rotary evaporator was used for drying all compounds.

General procedure for the synthesis of substituted 5-phenylfuran-2-carbaldehyde (2):[1]

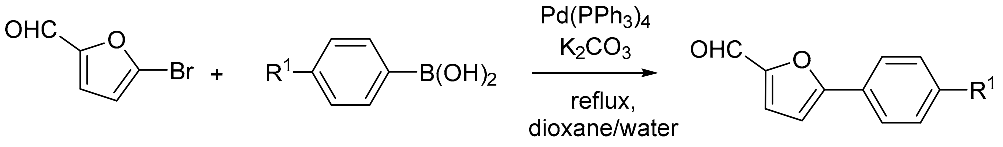

2

5-Bromofuran-2-carbaldehyde (400 mg, 2.28 mmol, 1 equiv.), (4-(trifluoromethyl)phenyl) boronic acid (430 mg, 2.28 mmol, 1 equiv.), potassium carbonate (3.16 g, 22.8 mmol, 10 equiv.), and tetrakis(triphenylphosphine)palladium(0) (266 mg, 0.23 mmol, 0.1 equiv.) were added to mixed solvents (6 mL dioxane and 4 mL water). The mixture was heated at reflux under argon protection for 6 h. The mixture was diluted with water (50 mL) and ethyl acetate (50 mL). The ethyl acetate layer was collected and washed with brine wash three times. The organic phase was dried by sodium sulfate and purified by flash chromatography, resulting in compound **2** as a yellow solid (yield: 55%).

General procedure for the synthesis of thiazolidine-2,4-dione:[2]

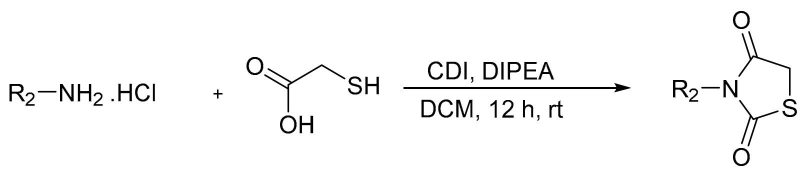

1b

A mixture of amine HCl salt (1 mmol, 1 equiv.) and DIPEA (3 mmol, 3 equiv.) in DCM (2 mL) was stirred for 10 min. at rt. To the resultant homogeneous solution was added thioglycolic acid (1 mmol, 1 equiv.) and 1,1'-carbonyldiimidazole (2 mmol, 2 equiv.), and the reaction mixture

was stirred at rt for 12 h. The mixture was diluted with DCM (50 mL) and saturated $NaHCO_3$ solution. The organic layer was separated and washed with brine, dried over Na_2SO_4, and concentrated in the rotary evaporator. Purification of the resultant residue by silica gel chromatography (EtOAc/Hexane) afforded the desired thiazolidine-2,4-dione (60-88%).

General procedure for the synthesis of thiazolidine-2,4-dione:[3]

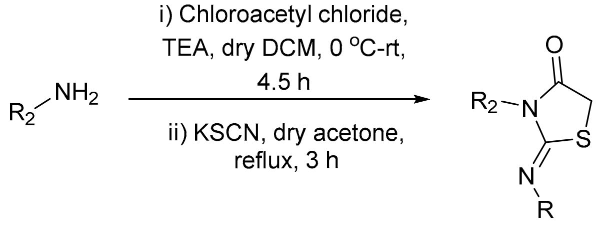

1c

To a solution of amine (1 mmol, 1 equiv.) in 10 mL of anhydrous DCM was added 2-chloroacetyl chloride (1.2 mmol, 1.2 equiv.) and Et_3N (0.1 mmol, 0.1 equiv.) at 0 °C. The resulting mixture was allowed to warm to room temperature and stirred for 4.5 h. The reaction was quenched by water and the water layer washed with ethyl acetate (3 x 50 ml). The combined organic phase was washed with brine, dried over Na_2SO_4, and concentrated in vacuo to afford the crude *N*-substituted-2-chloroacetamide. A mixture of the crude intermediate (1 mmol, 1 equiv.), KSCN (1.5 mmol, 1.5 equiv.), and dry acetone (5 mL) was stirred at reflux 3 h. Excess acetone was removed in vacuo and the residue was stirred with water (10 mL) for 1 h. The solid was filtered, washed with water, dried, and crystallized from EtOH or through flash chromatography to afford the compound **1c** (30-81%) as a solid.

General procedure for the synthesis of BW-FI-101-141:[3]

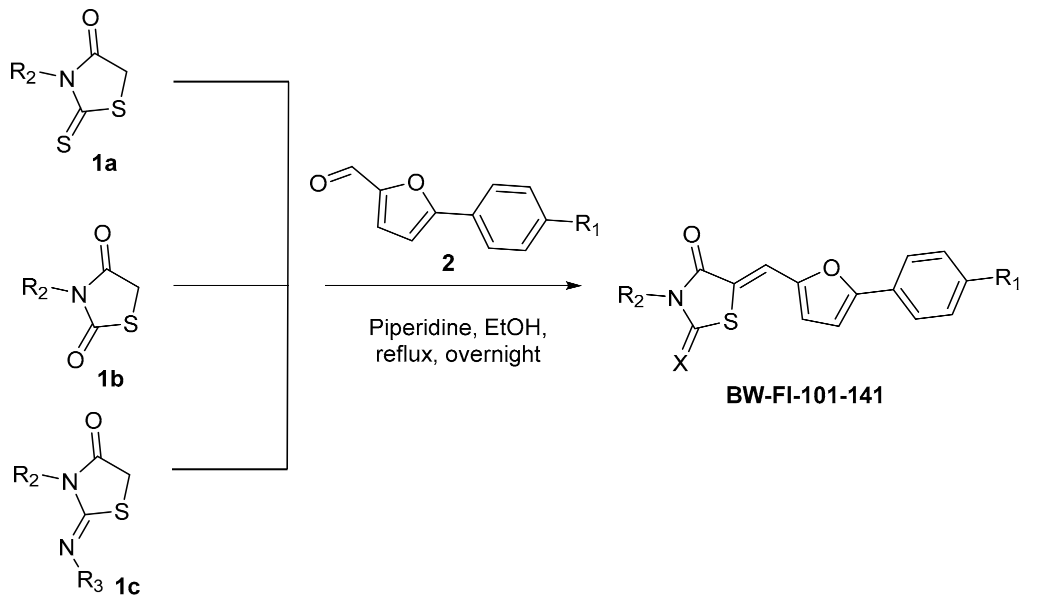

A mixture of **1a**, **1b,** or **1c** (1 mmol, 1 equiv.), aldehyde **2** (1.2 mmol, 1.2 equiv.), piperidine (1.2 mmol, 1.2 equiv.), and 1 mL of EtOH was stirred at reflux overnight. The reaction mixture was cooled to room temperature and the solid precipitates were collected by filtration, washed with EtOH, and dried in vacuo to afford **BW-FI-101-141** (59-94%) as a solid.

BW-FUI-106: Light Brown solid (71 % Yield), ^{1}H NMR ($CDCl_3$) δ 8.57 (d, *J* = 4.6 Hz, 1H), 7.86 (d, *J* = 8.2 Hz, 1H), 7.74 (d, *J* = 8.3 Hz, 1H), 7.66 (s, 1H), 7.24 (d, *J* = 5.8 Hz, 1H), 6.98 (d, *J* = 3.7 Hz, 1H), 6.95 (d, *J* = 3.7 Hz, 1H), 4.18 – 3.93 (m, 1H), 3.16 – 2.93 (m, 1H). ^{13}C NMR ($CDCl_3$) δ 168.4, 165.6, 156.3, 149.7, 149.5, 147.0, 126.1, 126.1, 124.6, 124.3, 120.3, 119.2, 119.0, 110.2, 41.6, 33.0.

BW-FUI-109: Brown solid (78 % Yield), ^{1}H NMR ($CDCl_3$) δ 8.57 (d, *J* = 4.6 Hz, 2H), 7.86 (d, *J* = 8.2 Hz, 2H), 7.74 (d, *J* = 8.3 Hz, 2H), 7.66 (s, 1H), 7.24 (d, *J* = 5.8 Hz, 2H), 6.98 (d, *J* = 3.7 Hz, 1H), 6.95 (d, *J* = 3.7 Hz, 1H), 4.09 – 4.02 (m, 2H), 3.09 – 3.01 (m, 2H). ^{13}C NMR ($CDCl_3$) δ 168.5, 166.0, 156.0, 149.9, 126.1, 126.1, 126.0, 126.0, 124.5, 119.8, 119.7, 118.6, 110.1, 55.4, 54.9, 38.8, 28.0.

BW-FI-112: Yellow solid (50% yield), ^{1}H NMR (DMSO-*d₆*) δ 9.81 (s, 1H), 8.48 (d, *J* = 4.4 Hz, 1H), 8.01 (d, *J* = 8.1 Hz, 2H), 7.86 (d, *J* = 8.1 Hz, 2H), 7.70 (t, *J* = 7.5 Hz, 1H), 7.51 – 7.43 (m, 2H), 7.29 – 7.19 (m, 2H), 7.16 (d, *J* = 3.5 Hz, 1H), 4.11 (t, *J* = 7.3 Hz, 2H), 3.08 (t, *J* = 7.3 Hz, 2H). ^{13}C NMR (DMSO-d_6) δ 165.8, 158.6, 154.5, 153.3, 150.9, 149.5, 137.0, 133.2, 128.8, 128.5, 126.5, 126.5, 125.9, 124.8, 123.6, 123.1, 122.1, 121.8, 119.5, 114.7, 112.2, 41.6, 35.2. HRMS (ESI) m/z calcd for $C_{22}H_{17}F_3N_3O_2S$ $[M+H]^+$ 444.0994, found 444.1008.

BW-FUI-113: Yellow solid (66% yield), ^{1}H NMR (DMSO-d_6) δ 9.79 (s, 1H), 8.48 (d, *J* = 4.5 Hz, 1H), 8.04 (d, *J* = 8.2 Hz, 2H), 7.87 (d, *J* = 8.3 Hz, 2H), 7.76 (s, 1H), 7.58 (s, 1H), 7.48 (d, *J* = 3.6 Hz, 1H), 7.29 (d, *J* = 8.0 Hz, 2H), 7.20 (d, *J* = 3.6 Hz, 1H), 5.07 (s, 2H). ^{13}C NMR (DMSO-d_6) δ 166.1, 155.5,

154.6, 153.3, 150.9, 149.5, 137.2, 133.1, 128.9, 128.6, 126.6, 125.8, 124.8, 123.1, 122.8,121.8, 121.4, 119.7, 115.1, 112.2, 46.2.HRMS (ESI) m/z calcd for $C_{22}H_{15}F_3N_3O_2S$ [M+H]$^+$ 430.0837, found 430.0845.

BW-FUI-124: Yellow solid (59% Yield) ^{1}H NMR ($CDCl_3$) δ 7.86 (d, *J* = 8.2 Hz, 2H), 7.73 (d, *J* = 8.4 Hz, 2H), 7.62 (s, 1H), 6.96 (d, *J* = 3.7 Hz, 1H), 6.90 (d, *J* = 3.7 Hz, 1H), 4.26 (dd, *J* = 8.6, 5.9 Hz, 1H), 2.48 (d, *J* = 19.5 Hz, 2H), 2.28 – 2.18 (m, 2H), 1.81 – 1.72 (m, 1H), 1.69 – 1.51 (m, 4H), 1.36 (t, *J* = 8.6 Hz, 1H), 1.31 – 1.20 (m, 2H). ^{13}C NMR ($CDCl_3$) δ 168.9, 166.7, 155.8, 150.1, 126.1, 126.0, 124.5, 119.7, 119.4, 118.1, 110.1, 59.5, 41.7, 37.4, 36.4, 36.0, 29.3, 27.9.

BW-FI-127: Yellow solid (42% yield), ^{1}H NMR ($CDCl_3$) δ 8.57 (s, 2H), 7.86 (d, *J* = 7.8 Hz, 4H), 7.74 (d, *J* = 7.9 Hz, 4H), 7.63 (t, *J* = 7.2 Hz, 2H), 7.50 (s, 2H), 7.19 (dd, *J* = 18.7, 6.4 Hz, 4H), 6.94 (s, 2H), 6.82 (d, *J* = 2.6 Hz, 2H), 4.28 (t, *J* = 7.0 Hz, 4H), 3.48 (dd, *J* = 13.9, 6.8 Hz, 4H), 3.22 (t, *J* = 7.0 Hz, 4H), 2.22 (s, 4H), 1.30 (t, *J* = 7.0 Hz, 7H). ^{13}C NMR (DMSO-d_6) δ 165.6, 158.6, 154.7, 150.8, 149.5, 147.4, 136.9, 133.0, 128.8, 128.5, 126.7, 126.7, 125.9, 124.9, 123.7, 123.2, 122.1, 119.9, 119.8, 115.6, 112.1, 47.2, 42.5, 35.2, 16.4. HRMS (ESI) m/z calcd for $C_{24}H_{21}F_3N_3O_2S$ [M+H]$^+$ 472.1307, found 472.1325.

BW-FI-128: Yellow solid (69% yield), ^{1}H NMR ($CDCl_3$) δ 7.71 (d, *J* = 8.6 Hz, 2H), 7.50 (s, 1H), 7.45 (d, *J* = 8.6 Hz, 2H), 6.81 (dd, *J* = 13.7, 3.6 Hz, 2H), 3.38 – 3.32 (m, 1H), 3.31 (s, 3H), 2.37 (s, 1H), 2.23 (d, *J* = 3.5 Hz, 1H), 1.79 (ddd, *J* = 18.8, 10.6, 5.4 Hz, 2H), 1.70 – 1.60 (m, 2H), 1.50 (d, *J* = 9.9 Hz, 1H), 1.34 – 1.21 (m, 3H). ^{13}C NMR ($CDCl_3$) δ 166.5, 155.4, 150.2, 134.1, 129.2, 128.1, 125.5, 120.1, 117.6, 115.2, 108.6, 65.3, 44.3, 40.7, 36.0, 35.6, 29.4, 29.0, 26.6. HRMS (ESI) m/z calcd for $C_{22}H_{22}ClN_2O_2S$ [M+H]$^+$ 413.1091, found 413.1087.

BW-FI-129: Yellow solid (41% yield), ^{1}H NMR ($CDCl_3$) δ 7.86 (d, *J* = 8.2 Hz, 2H), 7.72 (d, *J* = 8.2 Hz, 2H), 7.51 (s, 1H), 6.94 (d, *J* = 3.6 Hz, 1H), 6.81 (d, *J* = 3.6 Hz, 1H), 3.92 (q, *J* = 7.0 Hz, 2H), 3.35 (d, *J* = 5.6 Hz, 1H), 2.38 (s, 1H), 2.22 (d, *J* = 2.9 Hz, 1H), 1.77 (dd, *J* = 7.0, 4.1 Hz, 2H), 1.68 - 1.45 (m, 4H), 1.28 - 1.22 (m, 6H). ^{13}C NMR ($CDCl_3$) δ 166.1, 154.6, 150.9, 132.8, 125.9, 125.9, 124.2, 121.2, 117.3, 114.9, 109.9, 65.4, 44.3, 40.6, 38.0, 36.0, 35.6, 29.7, 29.0, 26.5, 12.7. HRMS (ESI) m/z calcd for $C_{24}H_{24}F_3N_2O_2S$ [M+H]$^+$ 461.1511, found 461.1503.

BW-FI-133: Yellow solid (61% yield), ^{1}H NMR (DMSO-d_6) δ 8.48 (d, *J* = 4.6 Hz, 1H), 8.04 (d, *J* = 8.2 Hz, 2H), 7.91 (d, *J* = 8.3 Hz, 2H), 7.77 (s, 1H), 7.62 (s, 1H), 7.49 (d, *J* = 3.6 Hz, 1H), 7.32 – 7.22 (m, 3H), 5.07 (s, 2H), 3.47 – 3.40 (m, 2H), 1.15 (t, *J* = 7.2 Hz, 3H). ^{13}C NMR (DMSO-d_6) δ 166.0, 155.5, 154.8, 150.8, 149.5, 147.6, 137.2, 133.0, 129.1, 128.8, 128.5, 128.2, 126.7, 126.7, 125.9, 124.9, 123.2, 122.8, 121.3, 120.0, 119.8, 116.0, 112.1, 47.1, 47.1, 16.3. HRMS (ESI) m/z calcd for $C_{23}H_{19}F_3N_3O_2S$ [M+H]$^+$ 458.1150, found 458.1135.

BW-FI-134: Yellow solid (67% yield), ^{1}H NMR (DMSO-d_6) δ 8.47 (d, *J* = 3.7 Hz, 1H), 7.86 (d, *J* = 8.5 Hz, 2H), 7.76 (t, *J* = 7.7 Hz, 1H), 7.61 (d, *J* = 8.6 Hz, 3H), 7.34 (d, *J* = 3.6 Hz, 1H), 7.28 (d, *J* = 7.8 Hz, 2H), 7.20 (d, *J* = 3.6 Hz, 1H), 5.06 (s, 2H), 3.19 (s, 3H). ^{13}C NMR (DMSO-d_6) δ 166.0, 155.5, 155.5, 150.1, 149.6, 149.5, 137.3, 133.5, 129.8, 128.3, 126.1, 122.8, 121.3, 120.2, 118.9, 116.2, 110.6, 47.1, 31.1. HRMS (ESI) m/z calcd for $C_{21}H_{17}ClN_3O_2S$ [M+H]$^+$ 410.0730, found 410.0735.

BW-FI-135: Yellow solid (52.8% yield), ^{1}H NMR (CDCl$_3$) δ 7.71 (d, *J* = 8.5 Hz, 2H), 7.50 (s, 1H), 7.45 (d, *J* = 8.5 Hz, 2H), 6.81 (dd, *J* = 15.7, 3.6 Hz, 2H), 3.92 (q, *J* = 7.0 Hz, 2H), 3.34 (d, *J* = 5.2 Hz, 1H), 2.37 (s, 1H), 2.22 (d, *J* = 3.2 Hz, 1H), 1.83 – 1.73 (m, 2H), 1.60 (s, 4H), 1.35 – 1.19 (m, 6H). ^{13}C NMR (CDCl$_3$) δ 166.1, 155.3, 150.3, 134.1, 129.1, 128.2, 125.4, 120.5, 117.4, 115.0, 108.5, 65.3, 44.3, 40.6, 37.9, 36.0, 35.6, 29.7, 29.0, 26.5, 12.7. HRMS (ESI) m/z calcd for $C_{23}H_{24}ClN_2O_2S$ [M+H]$^+$ 427.1247, found 427.1263.

BW-FI-136: Yellow solid (50% yield), ^{1}H NMR (CDCl$_3$) δ 7.86 (d, *J* = 8.2 Hz, 2H), 7.72 (d, *J* = 8.3 Hz, 2H), 7.51 (s, 1H), 6.94 (d, *J* = 3.6 Hz, 1H), 6.81 (d, *J* = 3.6 Hz, 1H), 3.35 (d, *J* = 5.3 Hz, 1H), 3.30 (s, 3H), 2.38 (s, 1H), 2.23 (d, *J* = 3.3 Hz, 1H), 1.86 – 1.73 (m, 2H), 1.68 - 1.57 (m, 2H), 1.49 (dd, *J* = 12.2, 3.3 Hz, 1H), 1.27 (dd, *J* = 18.7, 9.7 Hz, 3H). ^{13}C NMR (CDCl$_3$) δ 166.4, 154.7, 150.8, 146.5, 132.7, 130.0, 129.7, 125.9, 125.9, 125.3, 124.2, 122.6, 120.9, 117.5, 115.0, 109.9, 65.4, 44.3, 40.6, 36.0, 35.6, 29.4, 29.0, 26.6. HRMS (ESI) m/z calcd for $C_{23}H_{22}F_3N_2O_2S$ [M+H]$^+$ 447.1354, found 447.1333.

BW-FI-137: Yellow solid (93% yield), ^{1}H NMR (CDCl$_3$) δ 7.74 – 7.69 (m, 2H), 7.46 – 7.39 (m, 3H), 6.83 (d, *J* = 3.6 Hz, 1H), 6.77 (d, *J* = 3.6 Hz, 1H), 3.28 (s, 3H), 2.21 (s, 3H), 2.06 (d, *J* = 2.4 Hz, 6H), 1.84 – 1.74 (m, 6H). ^{13}C NMR (CDCl$_3$) δ 165.5, 155.0, 150.3, 134.1, 129.1, 128.2, 125.4, 117.2, 114.1, 113.5, 108.5, 55.6, 42.6, 36.5, 29.8, 29.7. HRMS (ESI) m/z calcd for $C_{25}H_{26}ClN_2O_2S$ [M+H]$^+$ 453.1404, found 453.1416.

BW-FI-138: Yellow solid (75% yield), ^{1}H NMR (CDCl$_3$) δ 7.72 (d, *J* = 8.6 Hz, 2H), 7.58 (s, 1H), 7.45 (ddd, *J* = 32.3, 15.4, 8.2 Hz, 7H), 6.83 (q, *J* = 3.6 Hz, 2H), 3.38 (d, *J* = 5.4 Hz, 1H), 2.31 (s, 1H), 2.19 (d, *J* = 3.7 Hz, 1H), 1.76 (ddd, *J* = 12.2, 7.4, 1.8 Hz, 1H), 1.67 – 1.49 (m, 3H), 1.47 – 1.40 (m, 1H), 1.33 – 1.20 (m, 3H), 1.15 (d, *J* = 9.6 Hz, 1H). ^{13}C NMR (CDCl$_3$) δ 166.0, 155.5, 150.2, 146.1, 135.2, 134.2, 129.2, 128.7, 128.1, 128.0, 125.5, 119.7, 117.9, 115.8, 108.7, 65.6, 44.1, 40.4, 36.0, 35.6, 29.0, 26.5. HRMS (ESI) m/z calcd for $C_{27}H_{24}ClN_2O_2S$ [M+H]$^+$ 475.1247, found 475.1250.

BW-FI-139: Yellow solid (63% yield), ^{1}H NMR (CDCl$_3$) δ 7.73 (d, *J* = 7.3 Hz, 2H), 7.58 (s, 1H), 7.46 (s, 4H), 7.35 (d, *J* = 7.6 Hz, 2H), 6.85 (s, 2H), 3.37 (s, 1H), 2.31 (s, 1H), 2.17 (s, 1H), 1.81 – 1.71 (m, 1H), 1.66 – 1.48 (m, 5H), 1.42 (d, *J* = 10.7 Hz, 1H), 1.32 – 1.12 (m, 3H). ^{13}C NMR (100 MHz, CDCl$_3$) δ 165.9, 155.7, 150.1, 145.9, 134.3, 133.8, 133.5, 129.3, 129.2, 128.9, 128.1, 125.5, 119.2, 118.2, 116.1, 108.7, 65.6, 44.1, 40.3, 35.9, 35.6, 29.0, 26.5. HRMS (ESI) m/z calcd for $C_{27}H_{23}Cl_2N_2O_2S$ [M+H]$^+$ 509.0857, found 509.0865.

BW-FI-140: Yellow solid (90% yield), ^1H NMR ($CDCl_3$) δ 7.71 (d, *J* = 8.6 Hz, 2H), 7.49 (s, 1H), 7.45 (d, *J* = 8.6 Hz, 2H), 6.81 (dd, *J* = 15.8, 3.6 Hz, 2H), 3.83 (t, *J* = 7.2 Hz, 2H), 3.34 (d, *J* = 5.5 Hz, 1H), 2.37 (s, 1H), 2.21 (s, 1H), 1.82 – 1.69 (m, 4H), 1.52 – 1.44 (m, 1H), 1.26 (dd, *J* = 19.1, 9.7 Hz, 3H), 0.95 (t, *J* = 7.4 Hz, 3H). ^{13}C NMR ($CDCl_3$) δ 166.5, 155.3, 150.3, 134.1, 129.2, 128.1, 125.4, 120.2, 117.5, 115.0, 115.0, 108.6, 65.3, 44.4, 44.3, 40.5, 36.0, 35.6, 29.0, 26.5, 20.7, 11.3. HRMS (ESI) m/z calcd for $C_{24}H_{26}ClN_2O_2S$ $[M+H]^+$ 441.1404, found 441.1408.

BW-FI-141: Yellow solid (81% yield), ^1H NMR ($CDCl_3$) δ 7.70 (d, *J* = 8.5 Hz, 2H), 7.44 (d, *J* = 9.8 Hz, 3H), 6.79 (dd, *J* = 19.4, 3.6 Hz, 2H), 4.89 (dt, *J* = 13.6, 6.8 Hz, 1H), 3.30 (d, *J* = 5.5 Hz, 1H), 2.37 (s, 1H), 2.22 (d, *J* = 2.9 Hz, 1H), 1.81 – 1.74 (m, 2H), 1.60 (ddd, *J* = 13.9, 7.5, 3.5 Hz, 3H), 1.50 (d, *J* = 6.9 Hz, 6H), 1.42 (d, *J* = 6.3 Hz, 1H), 0.97 – 0.80 (m, 3H). ^{13}C NMR ($CDCl_3$) δ 166.2, 155.2, 150.4, 134.0, 129.1, 128.2, 125.4, 117.2, 114.9, 108.5, 65.8, 47.5, 44.3, 40.5, 36.1, 35.7, 29.7, 29.0, 26.5, 19.1, 19.0. HRMS (ESI) m/z calcd for $C_{24}H_{26}ClN_2O_2S$ $[M+H]^+$ 441.1404, found 441.1408.

References:

1) Zhang, Fengying; Yang, Yang; Chen, Zhuang; Hao, Junsong; Zhang, Peng; Mao, Jun; Zhang, Yu; Wang, Shoukai; Guo, Chunlong. Polyheterocyclic compound, its preparation method and application as PGR75 agonist, WO 2024/103400 A1.

2) Keqiang, ye.; Asparagine endopeptidase (aep) inhibitors, compositions, and uses related thereto U.S. Patent WO 2020/242933 A1, December 3, **2020**.

3) Wang, G., Wang, X., Yu, H. *et al.* Small-molecule activation of the TRAIL receptor DR5 in human cancer cells. *Nat Chem Biol* 2013, **9**, 84-89.

2. NMR Spectra:

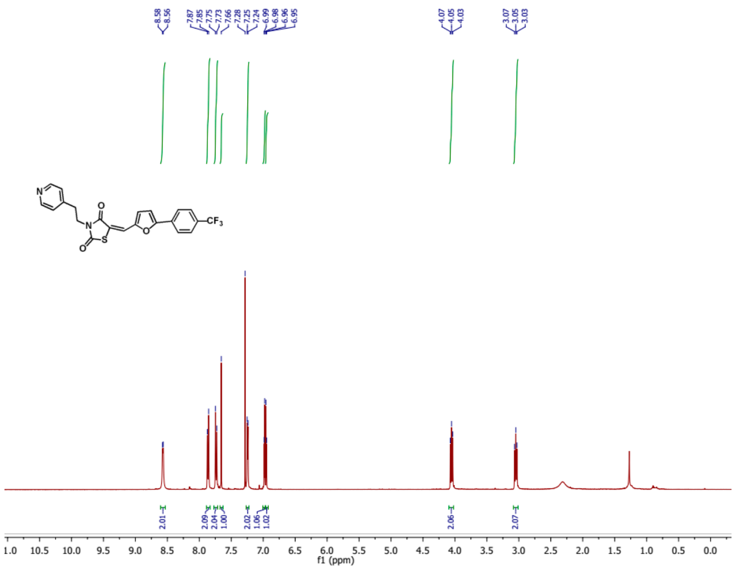

^{1}H NMR of BW-FI-106 in $CDCl_3$

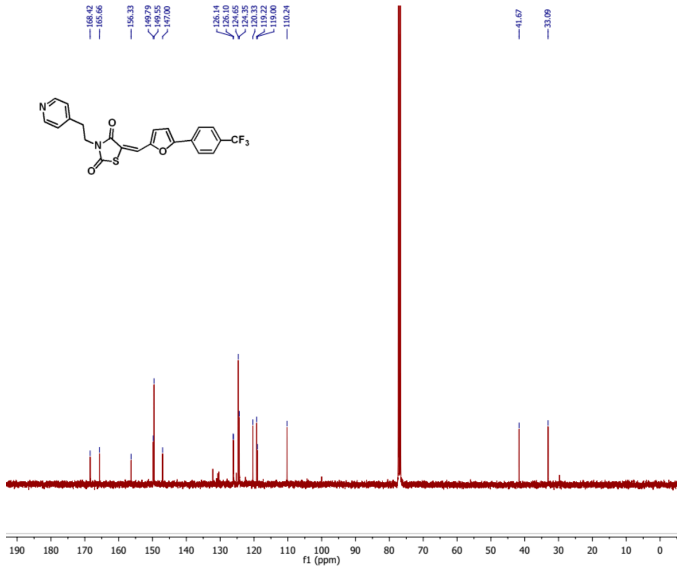

^{13}C NMR of BW-FI-106 in $CDCl_3$

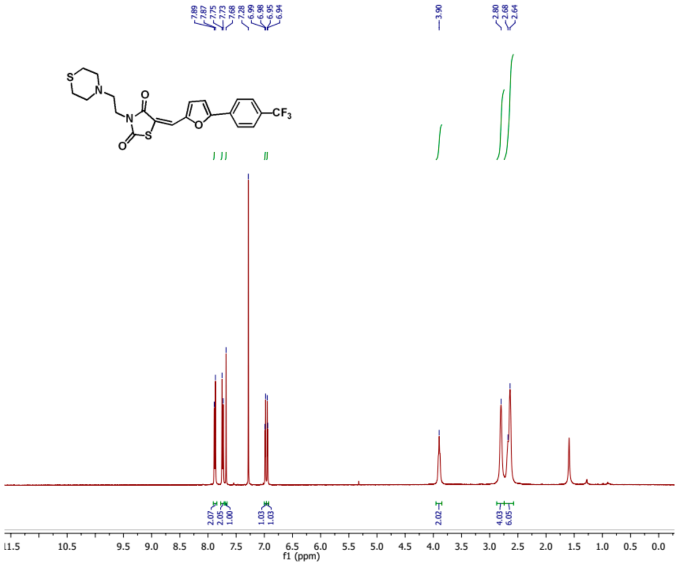

^{1}H NMR of BW-FI-109 in $CDCl_3$

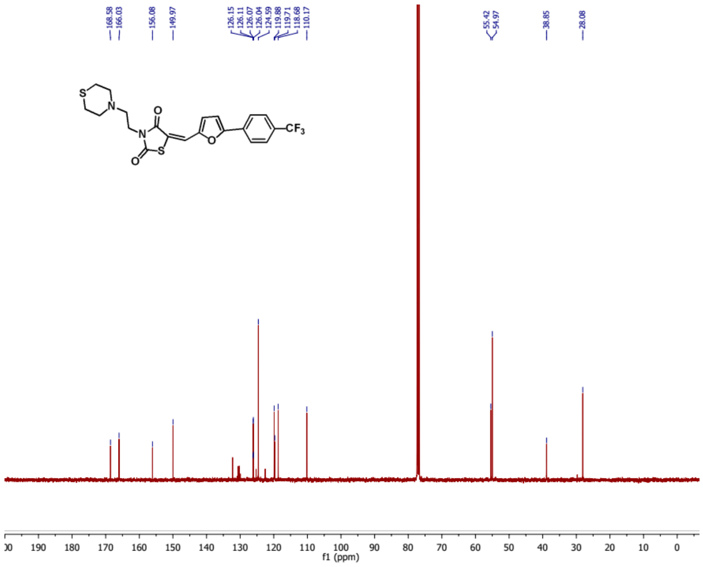

^{13}C NMR of BW-FI-109 in $CDCl_3$

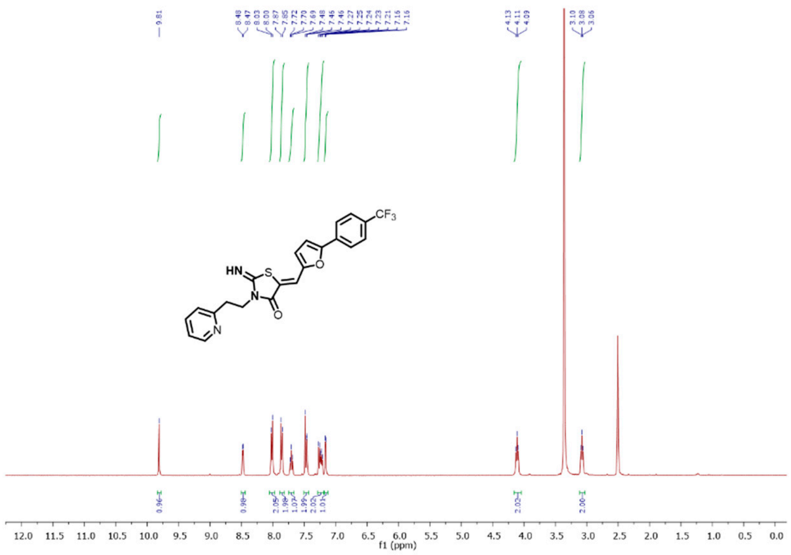

^{1}H NMR of BW-FI-112 in DMSO-d_6

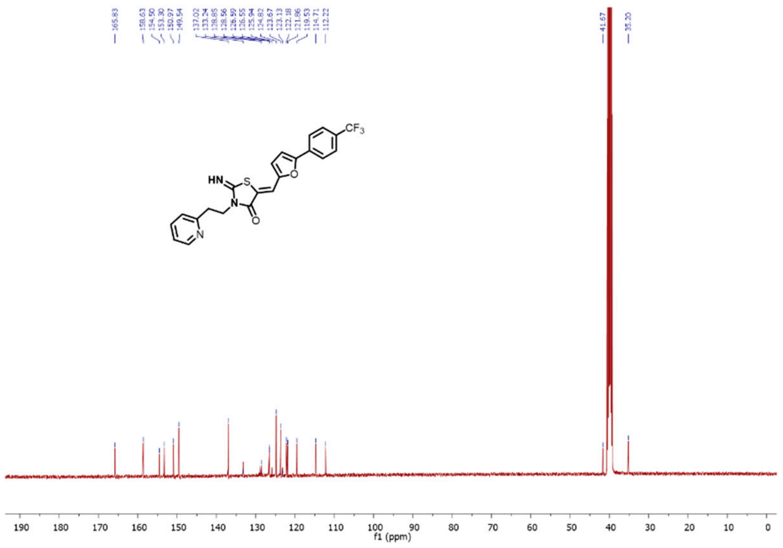

^{13}C NMR of BW-FI-112 in DMSO-d_6

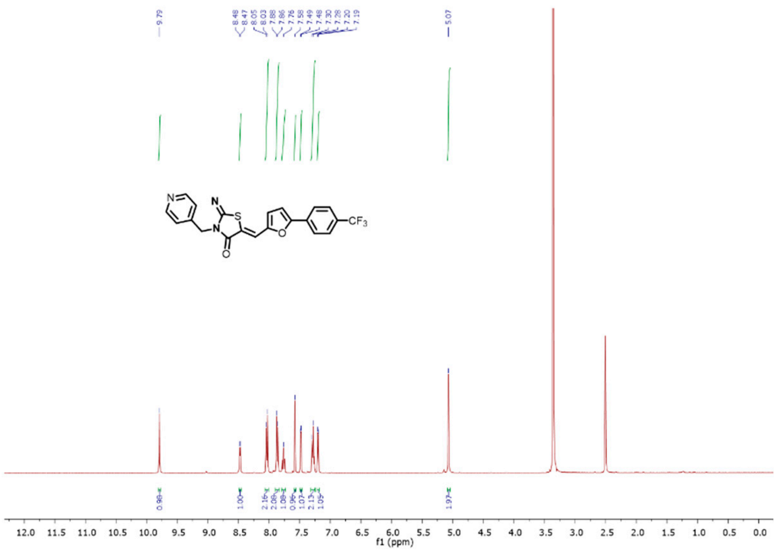

^{1}H NMR of BW-FI-113 in DMSO-d_6

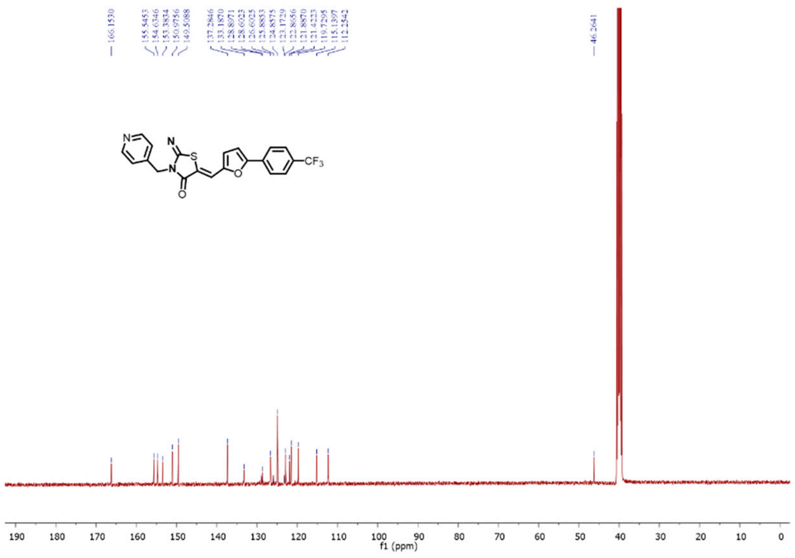

^{13}C NMR of BW-FI-113 in DMSO-d_6

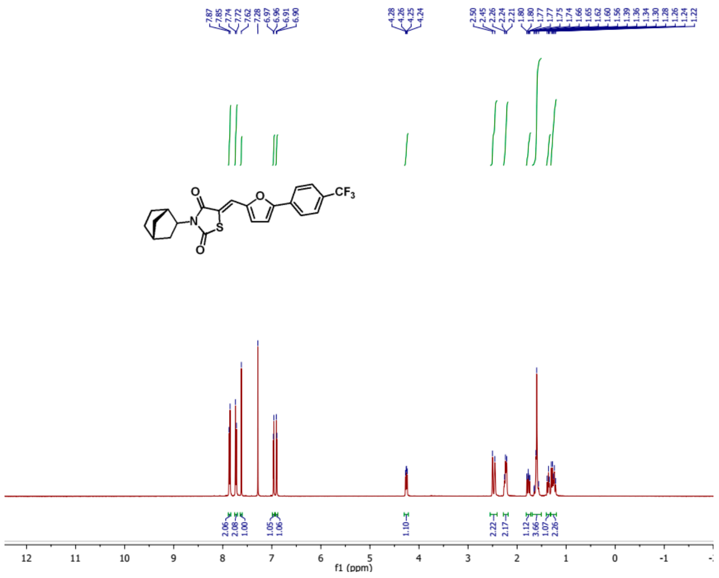

^{1}H NMR of BW-FI-124 in $CDCl_3$

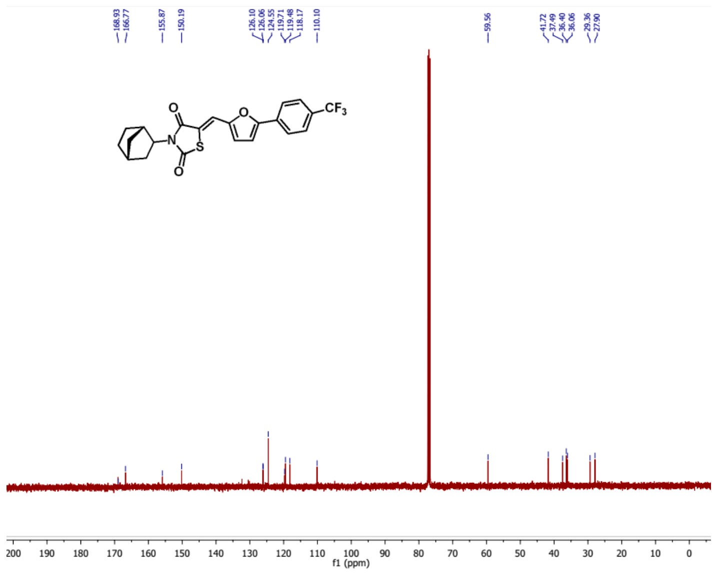

^{13}C NMR of BW-FI-124 in $CDCl_3$

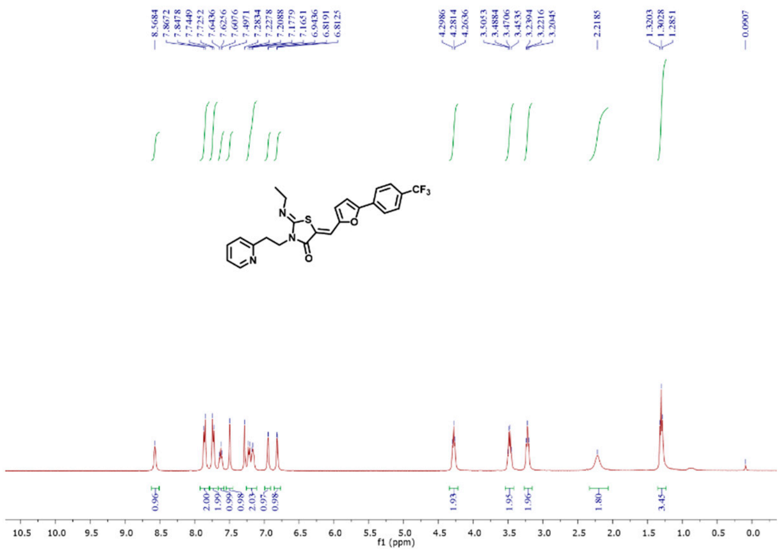

^{1}H NMR of BW-FI-127 in $CDCl_3$

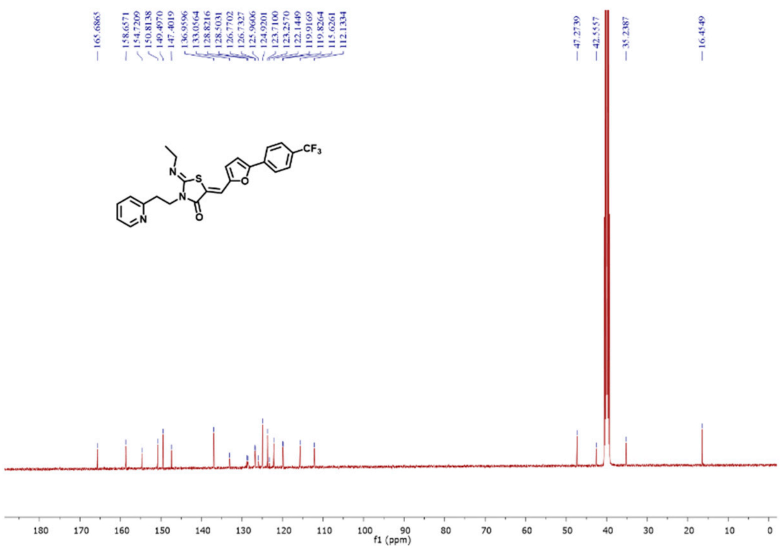

^{13}C NMR of BW-FI-127 in DMSO-d_6

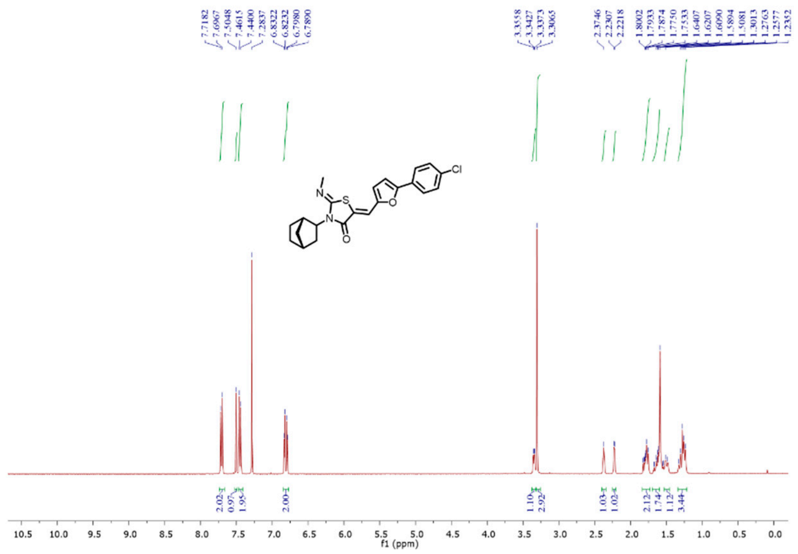

^{1}H NMR of BW-FI-128 in $CDCl_3$

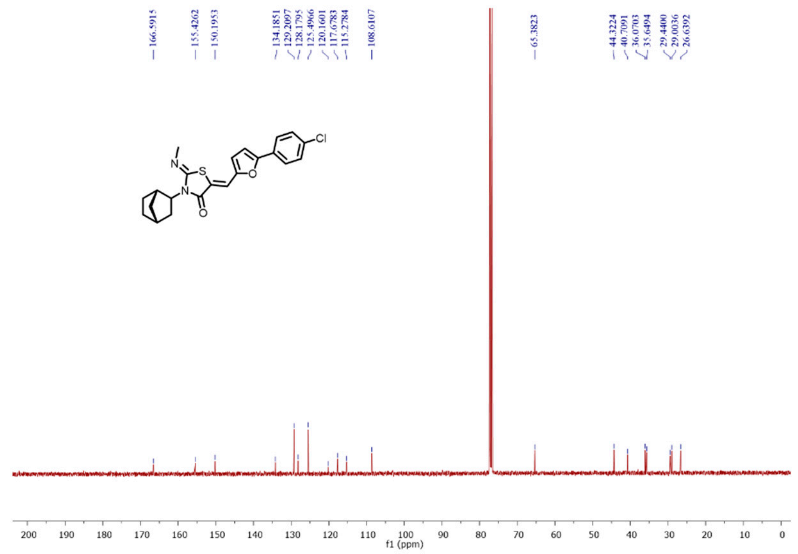

^{13}C NMR of BW-FI-128 in $CDCl_3$

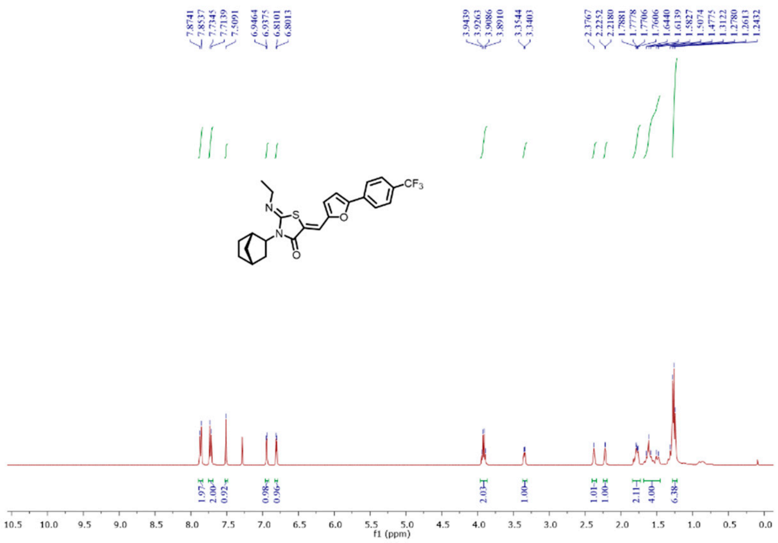

^{1}H NMR of BW-FI-129 in $CDCl_3$

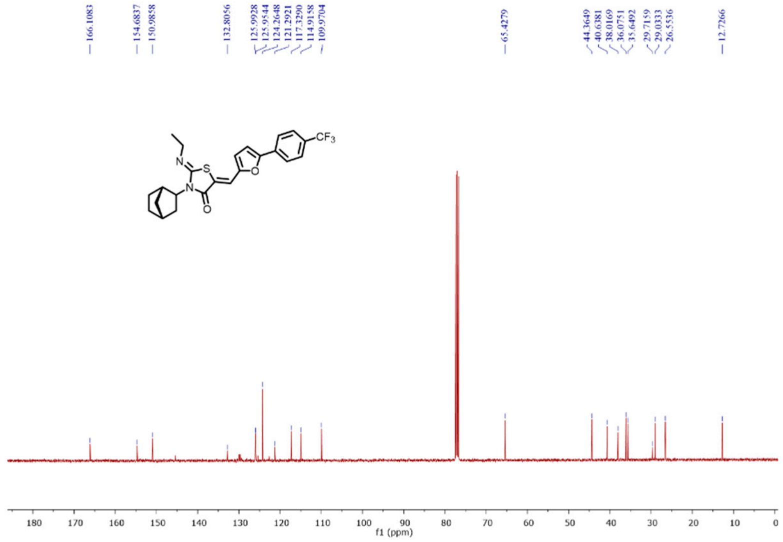

^{13}C NMR of BW-FI-129 in $CDCl_3$

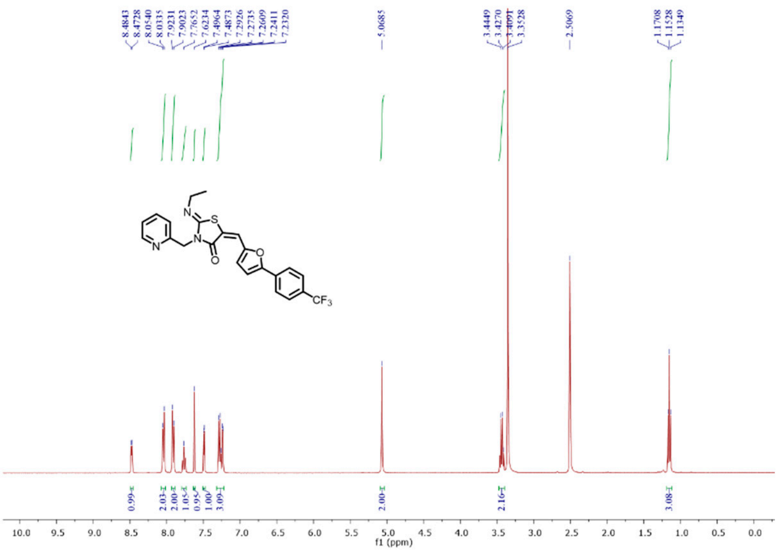

^{1}H NMR of BW-FI-133 in DMSO-d_6

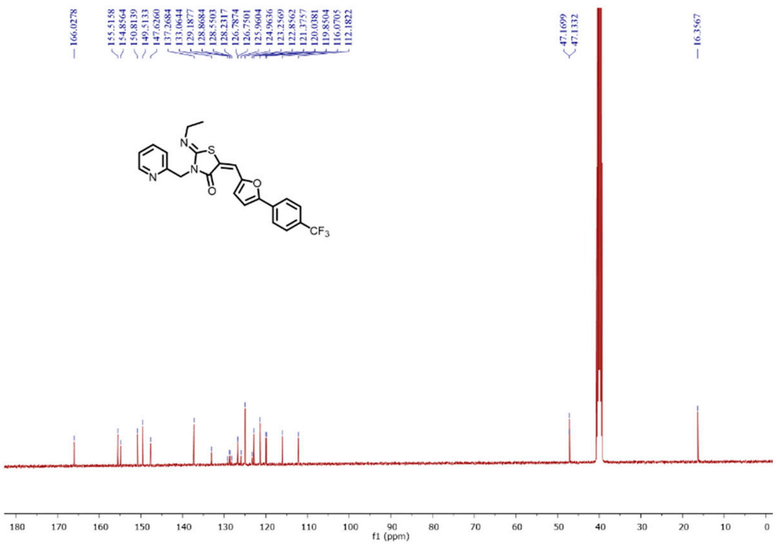

^{13}C NMR of BW-FI-133 in DMSO-d_6

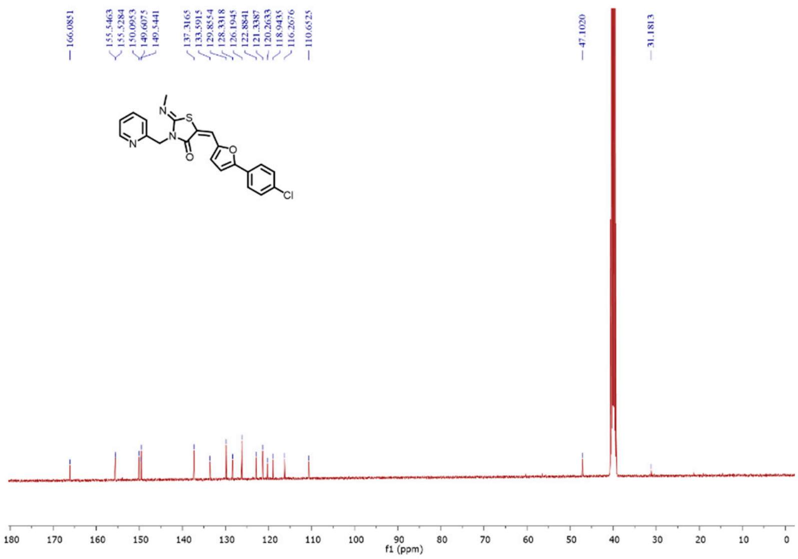

^{1}H NMR of BW-FI-134 in DMSO-d_6

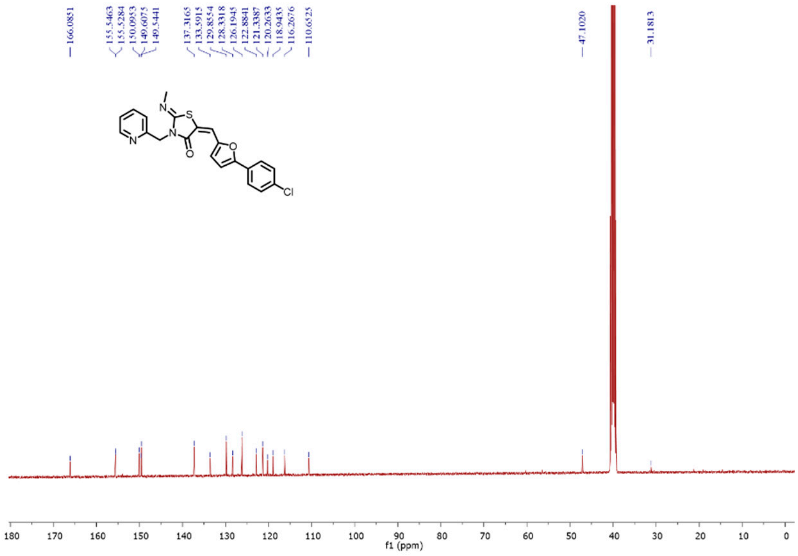

^{13}C NMR of BW-FI-134 in DMSO-d_6

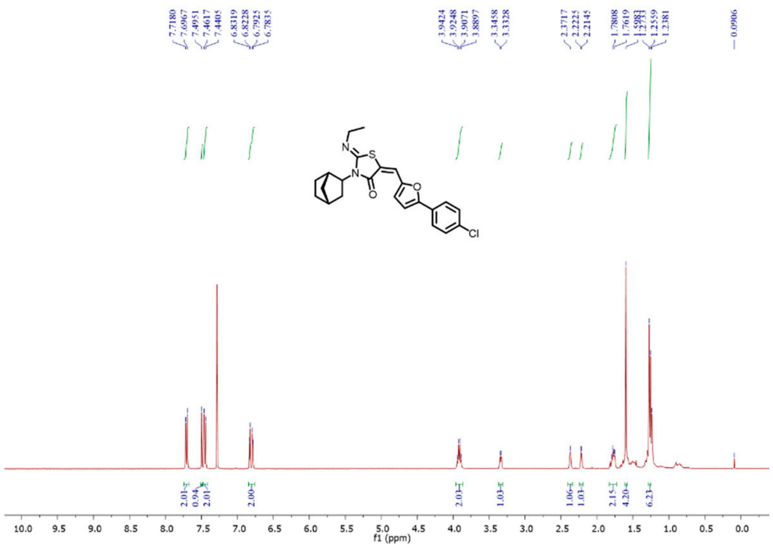

^{1}H NMR of BW-FI-135 in $CDCl_3$

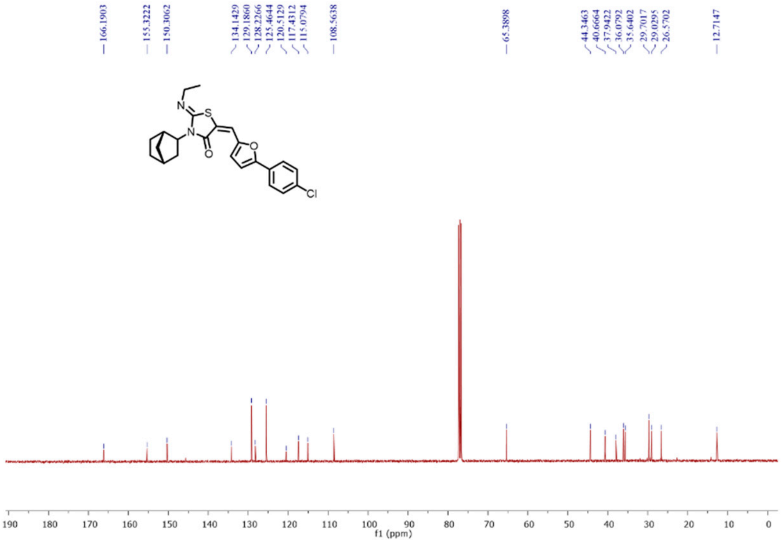

^{13}C NMR of BW-FI-135 in $CDCl_3$

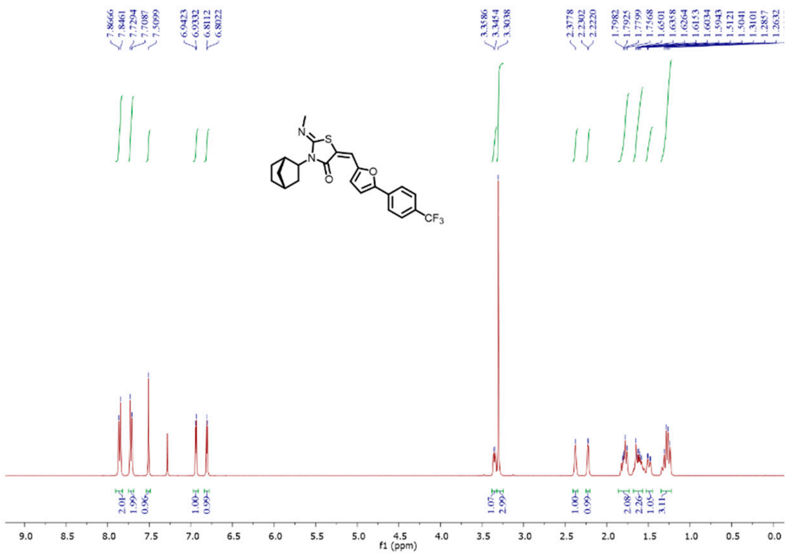

^{1}H NMR of BW-FI-136 in $CDCl_3$

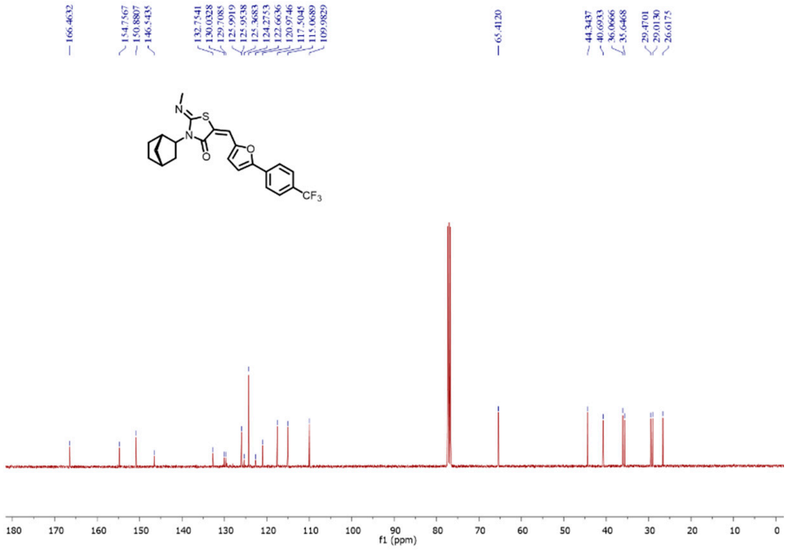

^{13}C NMR of BW-FI-136 in $CDCl_3$

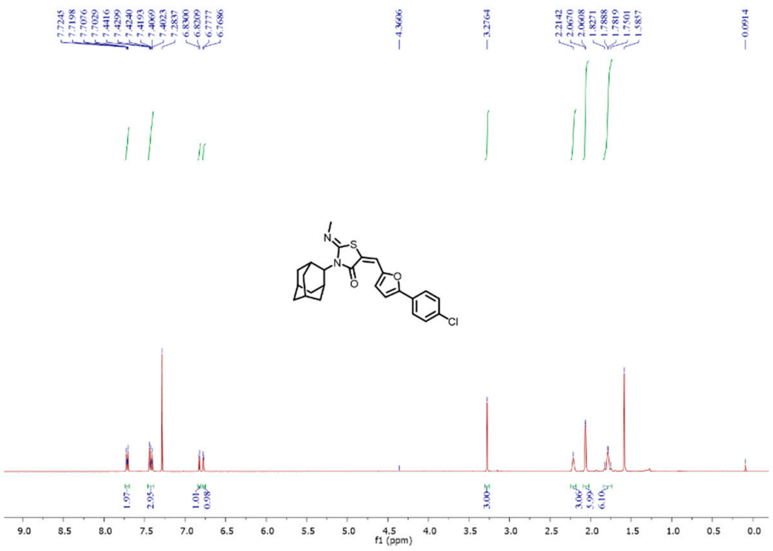

^{1}H NMR of BW-FI-137 in $CDCl_3$

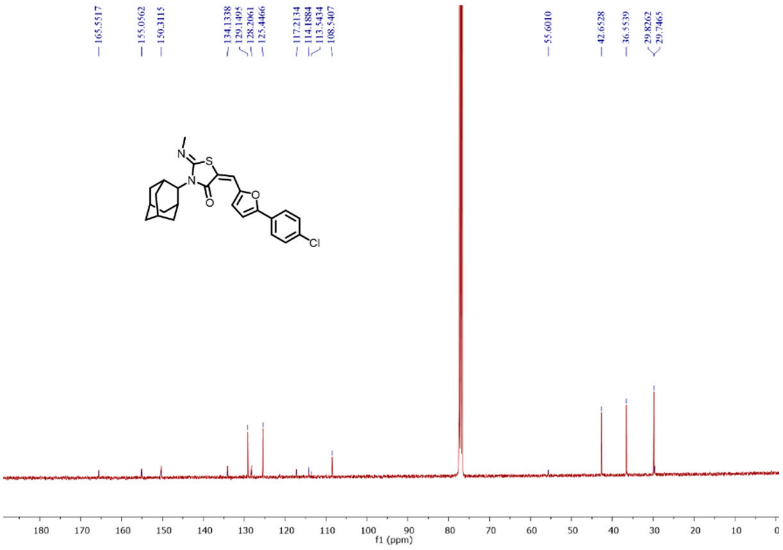

^{13}C NMR of BW-FI-137 in $CDCl_3$

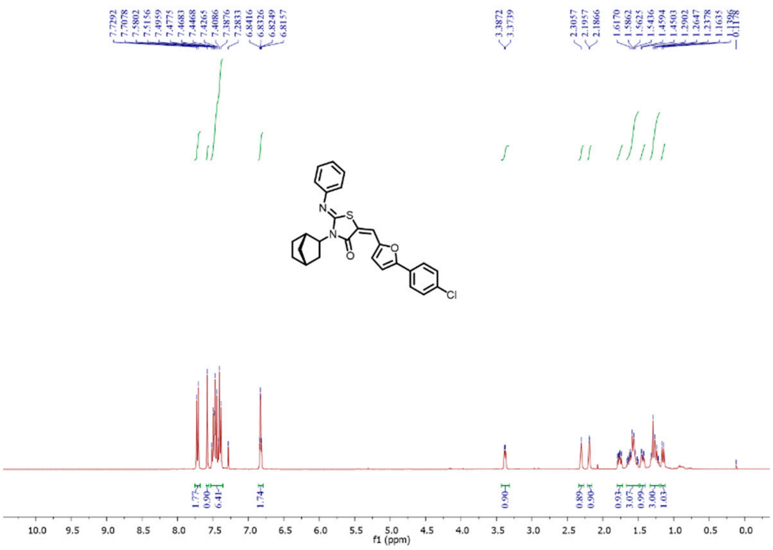

^{1}H NMR of BW-FI-138 in $CDCl_3$

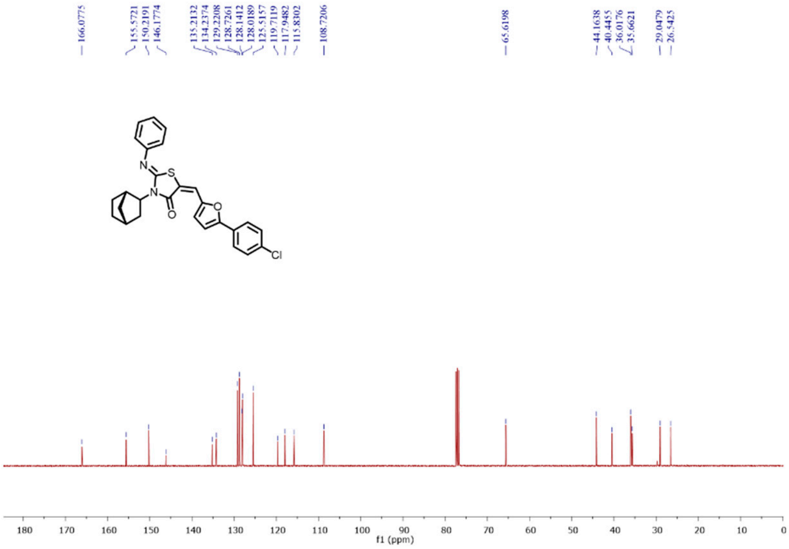

^{13}C NMR of BW-FI-138 in $CDCl_3$

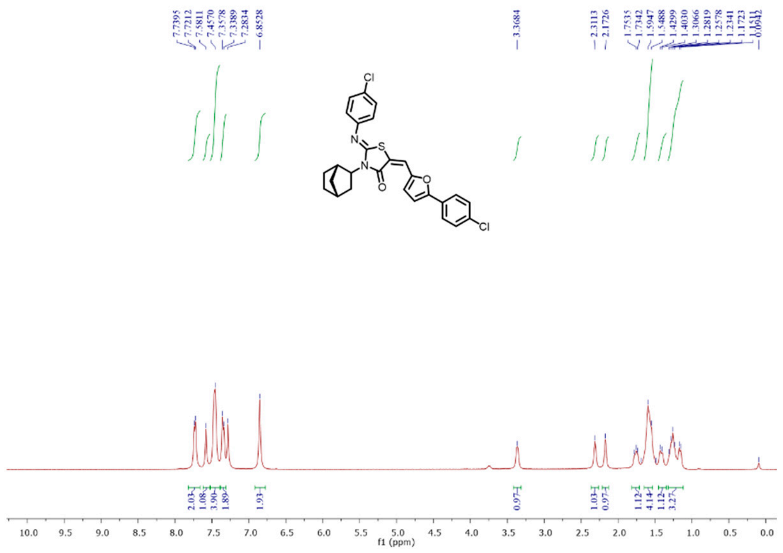

^{1}H NMR of BW-FI-139 in $CDCl_3$

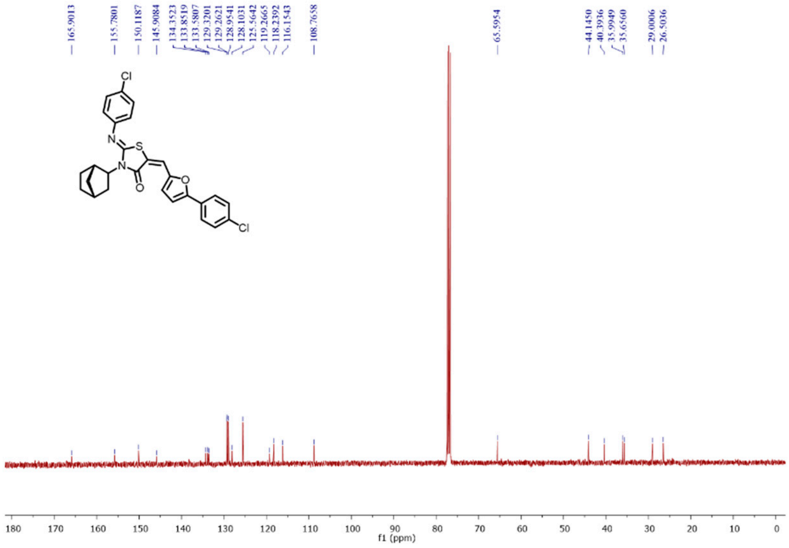

^{13}C NMR of BW-FI-139 in $CDCl_3$

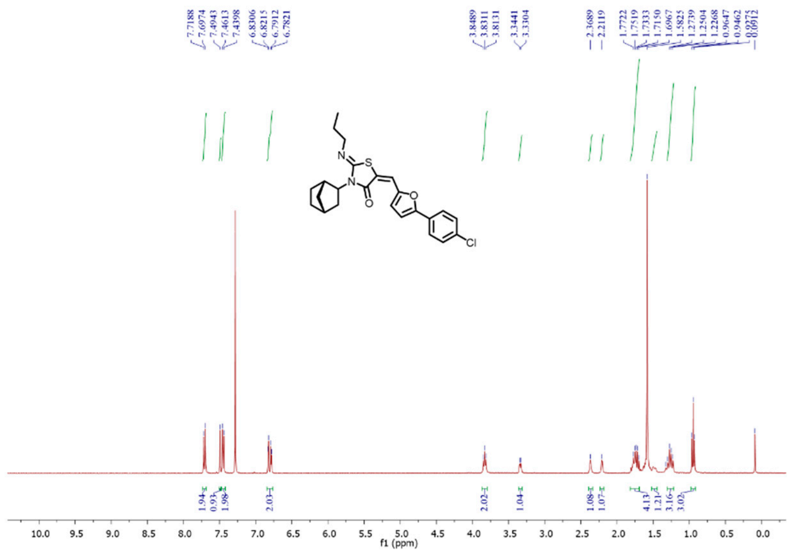

^{1}H NMR of BW-FI-140 in $CDCl_3$

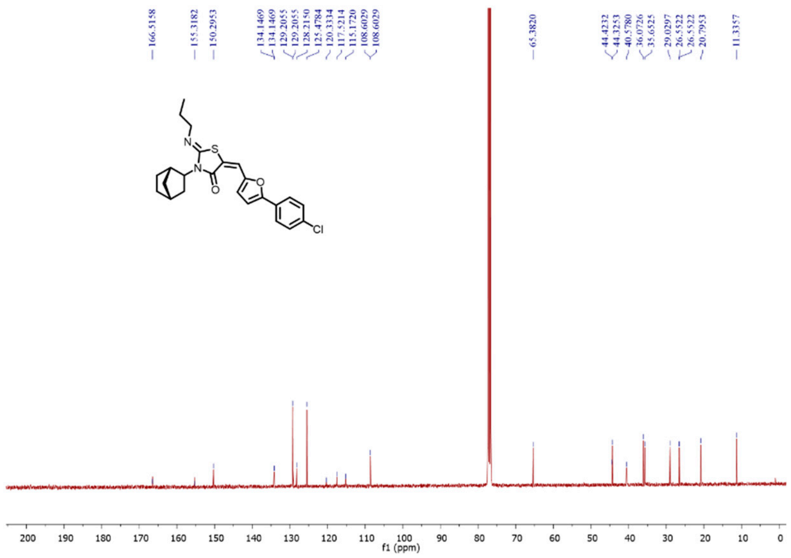

^{13}C NMR of BW-FI-140 in $CDCl_3$

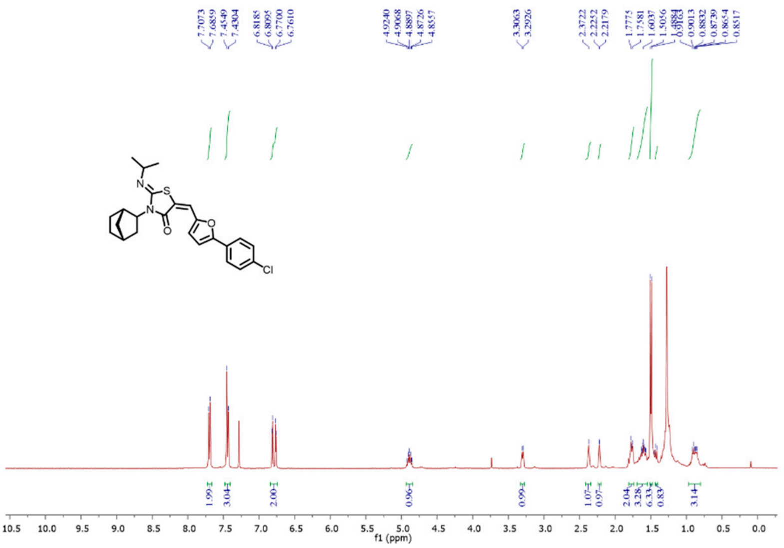

^1H NMR of BW-FI-141 in $CDCl_3$

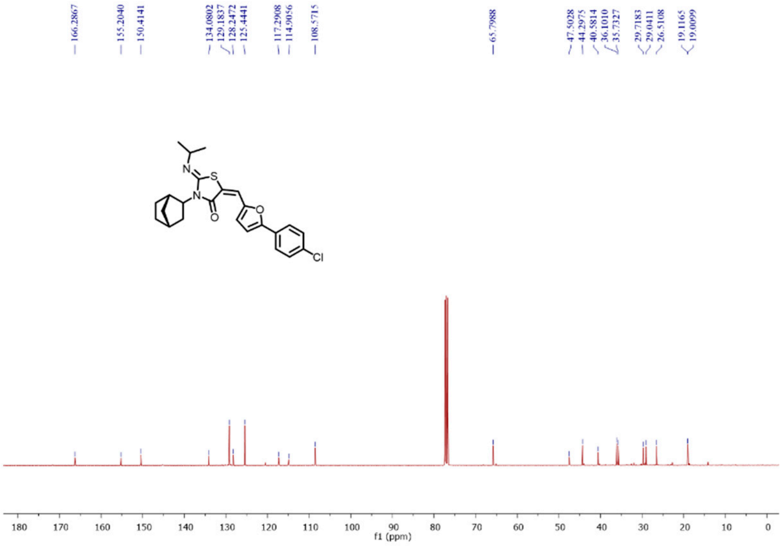

^{13}C NMR of BW-FI-141 in $CDCl_3$

3. **A structural classification that includes previously published analogues and the new synthesized analogues. The classification is based upon the common core structural features.**

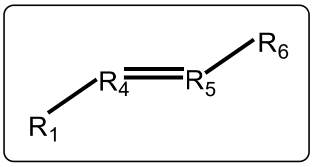

S. No.	Core Structure R_4	R_5	No. of analogues
**1.**	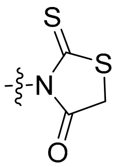 2-thioxothiazolidin-4-one	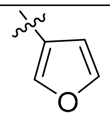	86
		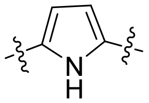	42
		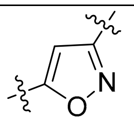	2
		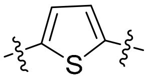	9
		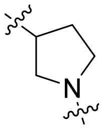	1
		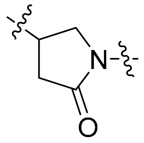	1
		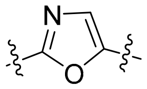	5
		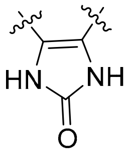	1
		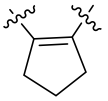	2
		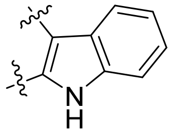	9
		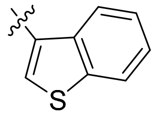 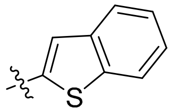	2
		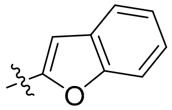	2

		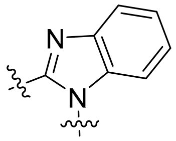	1
		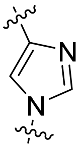	2
		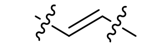	3
		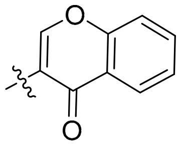	1
**2.**	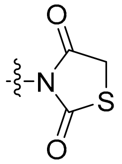<br>thiazolidine-2,4-dione	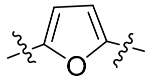	6
**3.**	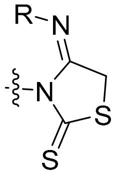<br>4-iminothiazolidine-2-thione	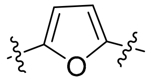	15
**4.**	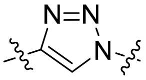<br>Triazole	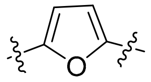	6

*Refer to the Excel sheet for overall structure for all compounds.

** For various substitutions on R^2 and R^3, R^1 and R^4, refer to the Excel sheet.
